# The gene regulatory basis of genetic compensation during neural crest induction

**DOI:** 10.1371/journal.pgen.1008213

**Published:** 2019-06-14

**Authors:** Christopher M. Dooley, Neha Wali, Ian M. Sealy, Richard J. White, Derek L. Stemple, John E. Collins, Elisabeth M. Busch-Nentwich

**Affiliations:** 1 Wellcome Sanger Institute, Wellcome Genome Campus, Hinxton, United Kingdom; 2 Department of Medicine, University of Cambridge, Cambridge, United Kingdom; University of Pennsylvania School of Medicine, UNITED STATES

## Abstract

The neural crest (NC) is a vertebrate-specific cell type that contributes to a wide range of different tissues across all three germ layers. The gene regulatory network (GRN) responsible for the formation of neural crest is conserved across vertebrates. Central to the induction of the NC GRN are *AP-2* and *SoxE* transcription factors. NC induction robustness is ensured through the ability of some of these transcription factors to compensate loss of function of gene family members. However the gene regulatory events underlying compensation are poorly understood. We have used gene knockout and RNA sequencing strategies to dissect NC induction and compensation in zebrafish. We genetically ablate the NC using double mutants of *tfap2a;tfap2c* or remove specific subsets of the NC with *sox10* and *mitfa* knockouts and characterise genome-wide gene expression levels across multiple time points. We find that compensation through a single wild-type allele of *tfap2c* is capable of maintaining early NC induction and differentiation in the absence of *tfap2a* function, but many target genes have abnormal expression levels and therefore show sensitivity to the reduced *tfap2* dosage. This separation of morphological and molecular phenotypes identifies a core set of genes required for early NC development. We also identify the 15 somites stage as the peak of the molecular phenotype which strongly diminishes at 24 hpf even as the morphological phenotype becomes more apparent. Using gene knockouts, we associate previously uncharacterised genes with pigment cell development and establish a role for maternal Hippo signalling in melanocyte differentiation. This work extends and refines the NC GRN while also uncovering the transcriptional basis of genetic compensation via paralogues.

## Author summary

Embryonic development is an intricate process that requires genes to be active at the right time and place. Organisms have evolved mechanisms that ensure faithful execution of developmental programmes even if genes fail to function. For example, in a process called genetic compensation, one or more genes become activated in response to loss of function of another. In this work we use the zebrafish model to investigate how two related genes, *tfap2a* and *tfap2c*, interact to ensure establishment of the neural crest, a vertebrate-specific cell type that contributes to many different tissues. Losing *tfap2a* activity causes mild morphological defects and losing *tfap2c* has no visible effect. Yet when both are inactive, embryos are severely abnormal due to lack of neural crest-derived tissues. Here we show that loss of *tfap2a* triggers upregulation of *tfap2c* which prevents the loss of neural crest tissue. However, the genes normally regulated by *tfap2a* respond differently to *tfap2c* allowing us to identify the first tier of the Ap2 network and new players in neural crest biology. Our work demonstrates that the expression signature of partial, but morphologically sufficient, genetic compensation provides an opportunity to dissect gene regulatory networks.

## Introduction

**Fig 1:**
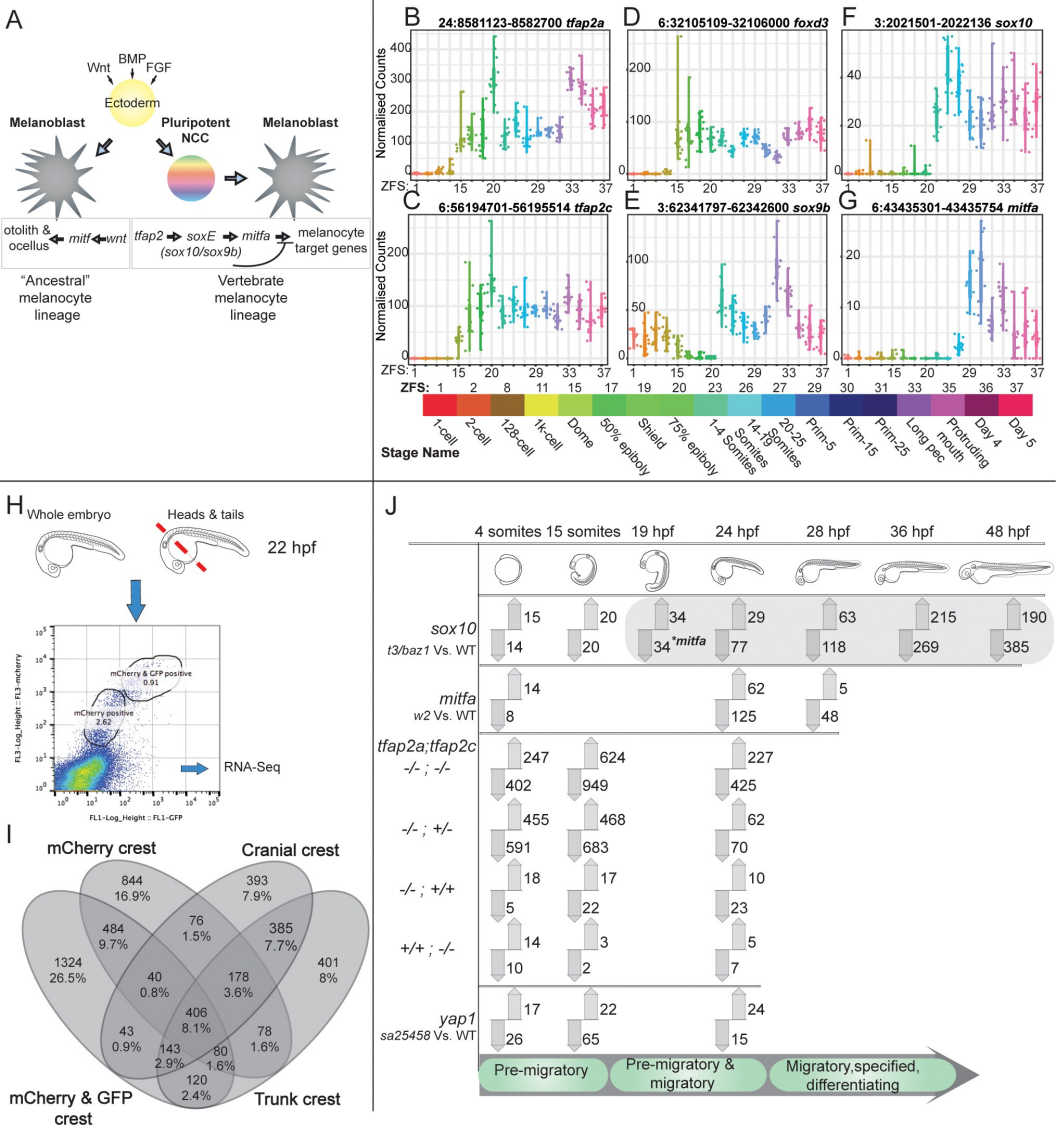
Analysis of the zebrafish NC GRN using gene expression data, knockouts and tissue-specific sequencing. (A) The NC is induced by different morphogens, for example Wnt, BMP and FGF acting on ectoderm. Non-vertebrate chordates lack NC cells but are capable of producing pigmented cells and otoliths via *mitf*. AP-2 and SoxE family genes are required in vertebrates to form the NC and these also contribute to the differentiation of specific NC tissues types. (B-G) 3’ end transcriptome sequencing (DeTCT) of six key neural crest transcription factors (*tfap2a*, *tfap2c*, *foxd3*, *sox9b*, *sox10*, *mitfa*) across 18 developmental time points covering zygote to 5 dpf. Normalised counts of individual embryos (dots) are plotted for each stage. The mapped GRCz10 genomic positions of each 3’ end are at the top of the plots next to the gene names. ZFS numbers are labelled with their corresponding stage names and representative colouring. (H) FACS of dissociated *sox10*:*mg* was sorted based on mCherry and GFP signals at 22–23 hpf and were either sorted as whole embryos or separated heads and tails. Multiple replicates of each cell population were harvested and sequenced via RNA-seq. (I) FACS transgenic populations were compared to non-transgenic populations using DESeq2 to produce gene enrichment lists for each population. The enriched gene lists for the mCherry and mCherry/GFP population from whole embryos and mCherry and/or GFP positive populations from the head or trunk were then compared to each other as a Venn diagram. (J) An overview of the transcriptomics loss of function analysis comparing mutants to WT siblings, using 3’ tag sequencing, carried out at stages of premigratory, migratory and differentiating neural crest cells. The phases of NC differentiation are noted at the bottom. Differentially expressed genes at adj. p-value <0.05 when compared to wild-type siblings are represented with up and down arrows for increased and decreased abundance, respectively. The *sox10* downstream target *mitfa* (*) is first detected as reduced (adj. p-value 0.019, log_2_ FC -1.775) at 19 hpf.

Development from a single fertilised cell to the complex adult form requires a simultaneously robust and plastic gene regulatory program. The neural crest is a transient pluripotent stem cell population capable of crossing germ layer boundaries and differentiating into highly diverse tissue types while migrating long distances in the developing embryo. The establishment of the neural crest and its subsequent tissue derivatives is specific to vertebrates and has played a fundamental role in their variation and evolutionary success [1–4]. Neural crest cells require a complex combination of external inductive signals such as Wnts, Fgfs, Notch/delta and Bmps (Fig 1A). These extrinsic signals can be considered the first phase of the neural crest gene regulatory network (GRN) followed by a second phase of tightly controlled intrinsic gene expression. In this context *foxd3* initially promotes neural crest fates by acting as a transcriptional repressor whereas later in development *foxd3* promotes neural crest fates as a transcriptional activator [5]. Two other intrinsic signals of fundamental importance for evolution and development of the neural crest that set vertebrates apart from other chordates such as amphioxus and tunicates are the *AP-2* and *SoxE* genes families [6–10].

Mutations in neural crest genes lead to disease in humans, highlighting the importance of this cell population for human health. Animal models faithfully recapitulate these defects demonstrating functional conservation. In humans and mice, mutations in *TFAP2A* lead to branchio-oculo-facial syndrome presenting as defects in cranial development and cranial closure [11,12]. Similarly, mutations in zebrafish *tfap2a* lead to craniofacial defects in addition to a reduction in melanocytes [13,14]. The Tfap2 family arose from a single gene in a chordate ancestor that underwent gene duplication resulting in five family members (*tfap2a*, *tfap2b*, *tfap2c*, *tfap2d* and *tfap2e*) in zebrafish. Removing combinations of *tfap2* family members results in a wide array of phenotypes. For example, the neural crest is completely ablated in *tfap2a*;*tfap2c* double homozygous zebrafish whereas there is a dramatic and specific reduction of melanocytes in *tfap2a*;*tfap2e* double homozygous zebrafish embryos [15–20]. Furthermore, melanomas, squamous cell carcinomas, most skin and breast cancers and a few cervical and urothelial cancers have strong nuclear immunoreactivity for TFAP2A. [21,22].

Haploinsufficiency in one of the AP-2 targets, the SoxE family member SOX10, results in Waardenburg syndrome; patients exhibit defects in the peripheral and enteric nervous systems and also pigmentation defects [23,24]. In zebrafish the known *SoxE* family members consist of *sox8a/b sox9a/b* and *sox10*. The expression of *sox10* is first detectable in premigratory neural crest cells and expression is maintained in certain neural crest linages, for example, glia, but reduced in many other neural crest-derived tissues in zebrafish [25–27] and mouse [28–30]. Following neural crest induction, *sox10* plays a vital role in the establishment of non-ectomesenchymal neural crest cells in zebrafish and driving *mitfa* expression. Knockouts in zebrafish *sox10* behave in a recessive manner and lead to the absence of enteric neurons, chromatophores, Schwann cells, sensory neurons and other trunk crest cell types [31,25,27]. Craniofacial features appear to be largely unaffected in zebrafish *sox10* mutants, which is thought to be due to compensation by *sox9b* in ectomesenchymal neural crest [32,33]. Mutations in *mitfa* lead to the total lack of body melanocytes in zebrafish due to a failure in melanocyte differentiation and as such *mitfa* is considered to be the melanocyte master regulator transcription factor [34].

Many crucial transcription factors involved in the neural crest GRN have been identified and studied in depth across a number of different species [10,35,36] and are largely conserved across vertebrates [37], but many of their downstream targets and interaction partners still remain to be elucidated. For example, AP-2A ChIP-seq analysis using human neural crest cells has identified over 4,000 potential AP-2A binding sites and established AP-2A as a chromatin initiating factor [38]. This large number of putative AP-2A downstream targets now requires functional validation.

In this work we use transcriptional profiling of zebrafish mutants in genes required at different levels of neural crest induction and differentiation to dissect the GRN downstream of *tfap2*. Specifically, by using individual genotyped embryos and many biological replicates, we can identify molecular neural crest signatures before a morphological phenotype arises. Stepwise genetic ablation of *tfap2* levels reveals in detail the gene regulatory basis of dose-dependent genetic compensation between two AP-2 paralogues. By analysing the dosage compensation we have identified a subset of genes required to rescue the neural crest. To validate a small subset of novel candidates emerging from this analysis we applied a reverse genetics approach to knock out genes of interest using both ENU and CRISPR/Cas9 mutagenesis [39–41]. Taken together, this work has identified early activation of members of the neural crest GRN and the core gene set underlying genetic compensation of *tfap2a* or *tfap2c* perturbations. Our screen has also identified novel downstream neural crest genes and a role for maternal expression of the Hippo signalling member *yap1* in the differentiation of melanocytes. All resources are publicly available and we envisage that this will lead to a deeper understanding of neural crest biology.

## Results

Our collection of mutations in previously well studied zebrafish mutants (*tfap2a*, *tfap2c*, *sox10* and *mitfa*) as well as a newly associated neural crest mutant (*yap1*–this study) encompasses an early undifferentiated, premigratory neural crest state through to terminal differentiation of different crest cell types, in particular the melanocytes.

### Neural crest GRN members are expressed at genome activation

Neural crest cells can be readily identified as the first somites begin to form, however it is not clear when the neural crest GRN becomes active in the zebrafish embryo. We used a wild-type developmental time course we had published previously [42] encompassing 18 stages from zygote to 5 dpf to identify the specific time points at which relevant transcripts are activated and their expression over time. In addition, the use of single embryos reveals the natural variation across individuals (Fig 1B–1G). In zebrafish, the genome first becomes transcriptionally active between the 1K-Cell and Dome stage [43–45]. A number of early neural crest transcription factors—*foxd3*, *tfap2a*, *tfap2c* - can be detected at the Dome stage. This is much earlier than neural crest cells are formed, but our data suggest that the top tier of the neural crest GRN has already commenced at these early developmental time points and is expressed in relevant ectoderm-forming regions (Fig 1B–1D) [2,46]. Their downstream targets *sox9b* and *sox10* begin to be expressed between 75% epiboly and when the first somites appear (Fig 1E and 1F). Both *sox9b* and *sox10* have been shown to be robust markers for premigratory neural crest cells in zebrafish [47].

### Identification of a neural crest–enriched gene set

We first created a catalogue of genes enriched in premigratory and differentiating neural crest cells as a reference set for the subsequent transcriptional analysis of the neural crest mutants. We used Fluorescence-Activated Cell Sorting (FACS) on dissociated cells from whole embryos of the *sox10*:*mg* line [48] at 22–23 hours post fertilisation (hpf). The transgenic reporter labels neural crest nuclei (mCherry) and crest cell membranes (GFP). At 22–23 hpf neural crest cells are migrating in a dorsal to ventral direction and their differentiation is more advanced at the rostral than caudal part of the embryo. We therefore reasoned that this stage would provide us with a comprehensive mixture of neural crest differentiation states. We compared transcripts detected in the transgenic neural crest cell populations to the non-crest cells using DESeq2 to produce neural crest-enriched gene sets (Fig 1I). For comparison to our whole embryo data we aggregated the resulting gene lists from the individual FACS experiments to produce a set of 4995 genes enriched in any FACS neural crest cell population (Table 1).

**Table 1:** Sequencing data and pairwise comparison gene lists.

Figure Location	Data Source	FigShare DOI
Fig 1I	FACS RNA-Seq gene lists	https://doi.org/10.6084/m9.figshare.6106082
Fig 1J	DeTCT gene lists (*sox10*, *mitfa*, *yap1*)	https://doi.org/10.6084/m9.figshare.6106091
Fig 2F	DeTCT *tfap2a;tfap2c* gene lists	https://doi.org/10.6084/m9.figshare.6106091
Fig 2F	UpSet gene subsets & ZFA Enrichment	https://doi.org/10.6084/m9.figshare.6170417
S2B Fig	RNA-Seq *tfap2a;tfap2c* gene lists	https://doi.org/10.6084/m9.figshare.6106079
Fig 4D	UpSet gene subset lists	https://doi.org/10.6084/m9.figshare.6170474
Fig 4I	Homer Results	https://doi.org/10.6084/m9.figshare.8131664
Fig 5B–5G	MCL cluster gene lists	https://doi.org/10.6084/m9.figshare.6170651
Fig 5B–5G	MCL cluster gene lists w/ genotype as sample name	https://doi.org/10.6084/m9.figshare.8131220

### *sox10* knockout mutants diverge from siblings transcriptionally at 19 hpf

In order to establish the initiation of the *sox10* GRN, a direct transcriptional target of *tfap2a;tfap2c*[49], we first created a transcriptional loss of function time course of *sox10* and its target, *mitfa*, by comparing gene expression of homozygous mutants and siblings. Zebrafish *sox10*^*t3/baz1*^ mutant embryos form premigratory neural crest cells in the trunk but these cells fail to migrate and properly differentiate while cranial crest remains largely unaffected [25]. Mutants of the *sox10*^*t3/baz1*^ downstream target *mitfa* have mostly correctly differentiated neural crest but specifically lack melanocytes of the body while showing mild differences in the numbers of the other two pigment cell types, xanthophores and iridophores [34].

Fig 1J is an overview of all experiments carried out using DeTCT (differential expression transcript counting technique) 3’ tag sequencing [50]. Although *sox10* is appreciably expressed at the 1–4 somites stage (~10 hpf) (Fig 1F) it is only at the 19 somite stage (19 hpf) where we detected a reduction in the abundance of one of its downstream targets, *mitfa*, in *sox10*^*t3/baz1*^ embryos (Fig 1J). The majority of genes appearing differentially expressed in the *sox10* 4 somite, 15 somite and 19 somite stages are localised on chromosome 3, the same chromosome as *sox10* (Fig 1J, Table 1). We also found similar signals for the *mitfa*^*w2/w2*^ mutants at 4 somites, with a very strong enrichment for chromosome 6 at 24 hpf (Table 1). This enrichment is either due to differential read mapping between the haplotypes owing to the high genetic variation in zebrafish [51] or reflects true expression differences between the two haplotypes, i.e. allele-specific expression [52]. When embryos homozygous for a specific genomic locus, in these cases the areas around the *sox10* or the *mitfa* mutation, are compared to embryos heterozygous and homozygous for the other haplotype, either possibility will cause an enrichment of DE genes on the chromosome carrying the mutation.

We next analysed enrichments of terms from the Zebrafish Anatomy Ontology (ZFA) associated with differentially expressed genes and plotted all time points with significant enrichments (S1 Fig). As expected, we found a strong and specific melanocyte signal in both mutants across all time points, with *sox10* mutants also showing a strong enrichment at 24 hpf for xanthophores and iridophores. By 36 hpf we also found an enrichment for the terms peripheral nervous system and nervous system which is consistent with an established role for *sox10* in peripheral nervous system development [25]. Previous data [25] and our developmental time course show that the expression of *sox10* begins early, following the establishment of the first neural crest cells at about 4 somites. It is only at the 19 somite stage, however, in which we detect the first molecular signal via the reduction of *mitfa* transcript, and only at 24 hpf do we see the first ZFA enrichments.

### Transcriptomic profiling of neural crest genetic ablation at three developmental stages using 3’ tag sequencing

Based on the wild-type expression of *tfap2a* and *tfap2c*, the morphological double mutant phenotype and the *sox10* molecular phenotype we chose three time points, 4 somites, 15 somites and 24 hpf, for the transcriptomic screen of *tfap2a;tfap2c* mutants. At the 4 somite stage pluripotent neural crest stem cells should be well established based on *snail1b* expression [33] and detectable with a whole embryo transcriptomic approach.

**Fig 2:**
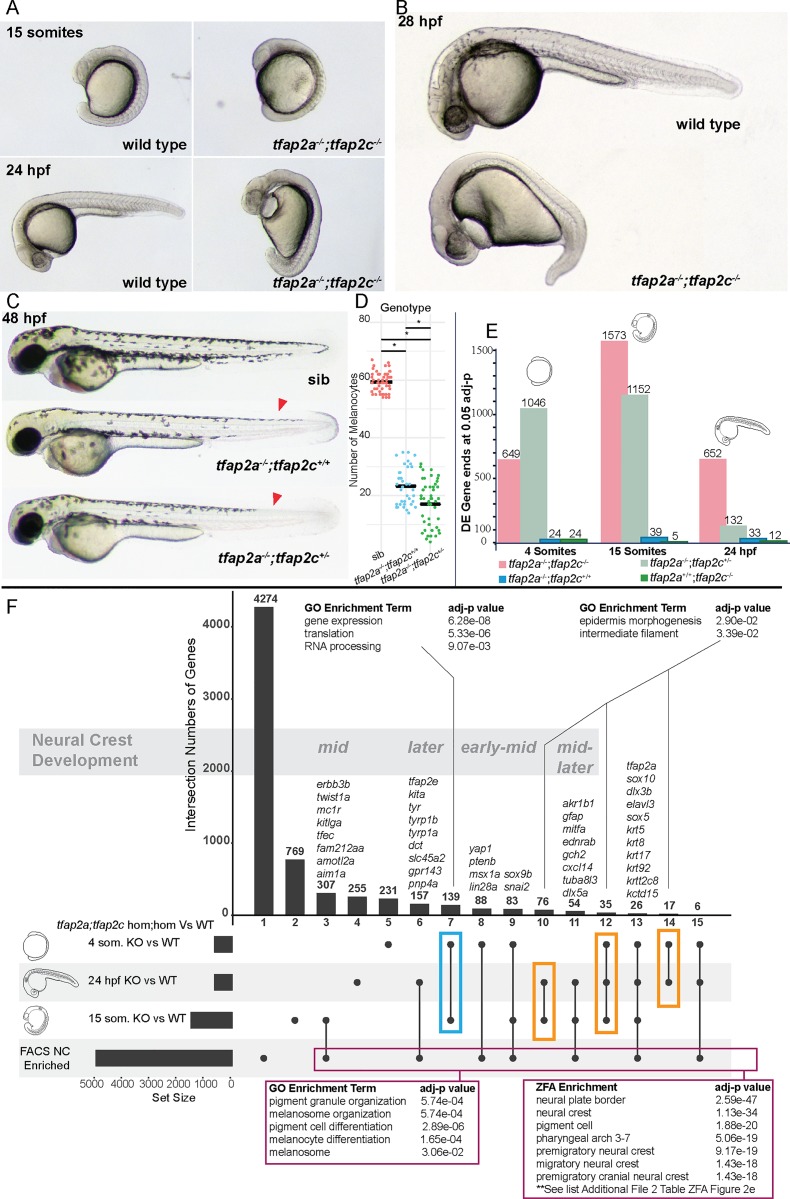
Molecular profiling of *tfap2a*;*tfap2c* mutants across multiple time points using 3’ tag sequencing. (A) *tfap2a*^*-/-*^;*tfap2c*^*-/-*^ mutants present the first morphological phenotypes at the 15 somite stage. (B) By 28 hpf the morphological phenotype leads to an overall dorsalised form, bifurcation of the forming eye, heart oedema, and complete lack of neural crest cells. All other genotypes appear normal. (C) At 48 hpf the previously described reduction of melanocytes can be noted in *tfap2a*^*-/-*^*;tfap2c*^*+/+*^ embryos and a modest reduction of melanocytes can be identified in the dorsal tail (red arrow heads) in *tfap2a*^*-/-*^*;tfap2c*^*+/-*^ mutants. (D) Quantification of melanocytes in the three corresponding genotypes at 36 hpf. (E) Chart indicating the number of differentially expressed gene 3’ ends identified with an adjusted p-value of <0.05 for each pairwise comparison of genotypes *tfap2a*^*-/-*^*;tfap2c*^*-/-*^, *tfap2a*^*-/-*^*;tfap2c*^*+/-*^, *tfap2a*^*-/-*^*;tfap2c*^*+/+*^ and *tfap2a*^*+/+*^*;tfap2c*^*-/-*^ to *tfap2a*^*+/+*^*;tfap2c*^*+/+*^ siblings at 4 somites, 15 somites and 24 hpf (F) An UpSet[53] diagram to compare multiple pairwise DE gene lists derived from the *tfap2a*^*-/-*^*;tfap2c*^*-/-*^ vs wild-type siblings (adj. p-value <0.05) for the 4 somite, 15 somite and 24 hpf stages and the list of neural crest-enriched genes derived from sorted neural crest cells at 22–23 hpf. The horizontal black bars represent the size of the gene lists. Individual subsets are marked with a black dot and overlaps with a connecting line. The number of genes in each subset is shown above each vertical bar. The vertical bars are numbered consecutively along the x-axis. GO/ZFA enrichment was carried out on the subset of the 4 and 15 somite stages (blue box), the subsets indicated with the orange boxes and on all genes contained in the neural crest FACS enrichment and in at least one of the three different double knockout time points (magenta box). The developmental time course nature of the data allows for the grouping of the subsets into timing based on neural crest development starting with *early* neural crest-specific gene expression and then moving towards *early-mid*, *mid*, *mid-later* and *later*. The complete list of the 26 genes in group 13 can be found in S2 Table.

To genetically ablate the neural crest, we created double carrier fish for *tfap2a*^*+/sa24445*^*;tfap2c*^*+/sa18857*^ (denoted as *tfap2a*^*+/-*^*;tfap2c*^*+/-*^ from here on) alleles, using mutants produced by the Zebrafish Mutation Project (ZMP https://www.sanger.ac.uk/resources/zebrafish/zmp/) [39]. We confirmed the phenotypes previously described in *tfap2a;tfap2c* depletion experiments [17,20]. Double homozygous embryos were indistinguishable from wild-type siblings at the 4 somite stage but were slightly elongated/dorsalised by the 15 somite stage and were clearly discernible by 24 hpf (Fig 2A and 2B). Notably, we also identified a specific pattern of reduction of dorsal tail melanocytes in *tfap2a*^*-/-*^*;tfap2c*^*+/-*^ embryos at 48 hpf (Fig 2C) in addition to the melanocyte reduction previously noted in *tfap2a*^*-/-*^ embryos which demonstrates a dosage effect of *tfap2c* heterozygosity on *tfap2a* homozygous mutants (Fig 2D). Conversely, all other genotypic combinations were indistinguishable from their wild-type siblings at 48 hpf with only *tfap2a*^*-/-*^ carriers progressing to present craniofacial defects at 72 hpf as previously described [16]. This phenotypic diversity shows that *tfap2a* and *tfap2c* do not act in a simple redundant fashion.

In light of the observed phenotypes stemming from a dosage effect of *tfap2c* heterozygosity in *tfap2a* homozygous mutants our primary aim was to systematically investigate the genetic interactions of *tfap2a* and *tfap2c*. We therefore sequenced up to 10 embryos for all 9 genotypes at the three different stages to enable comparison of all genotypic combinations. We crossed double heterozygous *tfap2a;tfap2c* parents and collected embryos at the three developmental time points as single embryos. Following nucleic acid extraction and genotyping, single embryos were processed and global mRNA transcript levels determined using 3’ tag sequencing (Fig 1J). After quality control and the removal of outlier samples we carried out pairwise analysis using DESeq2.

### Transcriptional phenotypes in *tfap2a* and *tfap2c* mutants differ greatly in magnitude when compared to their morphological outcomes

Our transcriptomic profiling uses whole embryos, therefore tissue-specific gene expression changes tend to be reflected in smaller log_2_ fold changes than would be expected from tissue dissection or FACS-derived cell populations. However, using high numbers of biological replicates enables us to faithfully detect smaller, but meaningful, effect sizes. We first assessed how the transcriptomes of the different genotypic conditions behaved across time. Comparing the absolute numbers of differentially expressed (DE) genes of the four most relevant knockout genotypes over the three developmental time points revealed three major findings (Fig 2E). Firstly, when compared to wild-type siblings, the number of genes differentially expressed in both *tfap2a* or *tfap2c* single homozygous embryos is very small in contrast to the double homozygous knockout and the *tfap2a*^*-/-*^*;tfap2c*^*+/-*^ mutants indicating genetic compensation. Secondly, despite the severe morphological phenotype of double mutants at 24 hpf the number of DE genes was less than half of that at the 15 somite stage. Conversely, while only beginning to display a mild morphological phenotype at 48 hpf, the *tfap2a*^*-/-*^*;tfap2c*^*+/-*^ mutants showed a strong molecular phenotype at 4 and 15 somites, with a longer DE list at 4 somites than the double mutants. This molecular signature was strongly diminished by 24 hpf. Taken together this demonstrates that the complexity of transcriptional changes is not necessarily mirrored in the morphological phenotype, and vice versa.

### Overlapping multiple expression profiles groups genes by biological function

Next we analysed the transcriptional profile of complete ablation of the neural crest in *tfap2a*^*-/-*^*;tfap2c*^*-/-*^ knockouts. A role for *tfap2a* has been previously described in both neural and non-neural ectoderm tissues which lead to the formation of the neural crest, epidermis, and cranial placodes [17,54,55]. To separate transcripts into subsets specific to the neural crest or the epidermis we filtered the DE genes from the three developmental time points in *tfap2a*^*-/-*^*;tfap2c*^*-/-*^ knockouts relative to wild-type siblings with the list of 4995 FACS-identified neural crest genes (Fig 2F). When all genes which appear in at least one of the developmental stages and the neural crest FACS list are analysed together with their associated GO terms, there is an enrichment for pigment cells and melanocytes but no other neural crest subtypes (magenta box Fig 2F). However, zebrafish anatomy enrichment (ZFA) returns a strong enrichment for the neural crest (Fig 2F, S1 Table). This finding highlights the current limitations of zebrafish GO annotation which has a bias for genes linked to pigmentation and lacks annotation for genes associated with earlier neural crest states.

A relatively small group of 26 genes (S2 Table) appearing in all four data sets included *tfap2a*, *sox10* and many keratins. This could potentially signify an epidermal/neural crest precursor cell type which is in the process of committing to one of the lineages.

Comparison of the three developmental time points places genes into “early,” “mid,” and “later” neural crest-specific groups. Each of these groups (Fig 2F) contain numerous examples of previously characterised neural crest-specific genes common across many studied species [37] which helps to validate this approach, but also many unannotated genes or genes previously not associated with the neural crest (Table 1).

The gene lists shared between the different stages but not found in the neural crest FACS data set (orange boxes Fig 2F) and their Gene Ontology (GO) term annotation revealed an enrichment for epidermal-related terms. Another subset from the 4 somite and 15 somite stages that is not present in the NC-enriched gene list is a group of genes enriched for *expression*, *translation* and *RNA processing* (blue box Fig 2F).

### *tfap2a;tfap2c* genetic compensation

**Fig 3:**
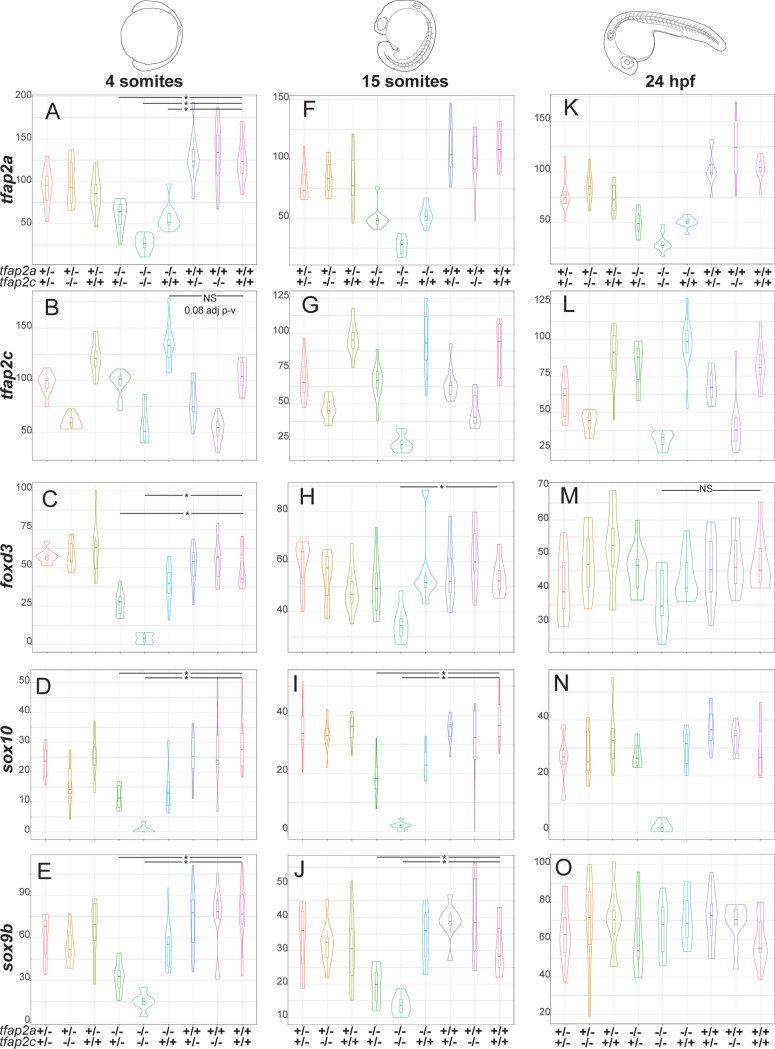
Expression of *sox10*, *sox9b* and *foxd3* in *tfap2a*;*tfap2c* mutants across three developmental time points. Normalised counts and gene name to the left of the violin plots and the corresponding genotypes for *tfap2a* and *tfap2c* at the bottom. All plots are ordered by the time points shown on the top of the figure. (A) At 4 somites levels of *tfap2a* are significantly lower than in wild-type siblings in all *tfap2a*^*-/-*^ genotypes. (B) Levels of *tfap2c* present at elevated levels in *tfap2a*^*-/-*^;*tfap2c*^*+/+*^ embryos when compared to wild-type siblings but fail the statistical cut-off (adj p-value 0.08). (C-E) Levels of *foxd3*, *sox10* and *sox9b* are significantly different in both *tfap2a*^*-/-*^;*tfap2c*^*+/-*^ and *tfap2a*^*-/-*^;*tfap2c*^*-/-*^ embryos but not in *tfap2a*^*-/-*^;*tfap2c*^*+/+*^. (F-G) At 15 somites the levels of *tfap2a* and *tfap2c* recapitulate trends observed at 4 somite stage. (H) Levels of *foxd3* are only significantly different in *tfap2a*^*-/-*^;*tfap2c*^*-/-*^ embryos when compared to wild-type siblings. (I-J) The levels of *sox10* and *sox9b* are both significantly different in *tfap2a*^*-/-*^;*tfap2c*^*+/-*^ and *tfap2a*^*-/-*^;*tfap2c*^*-/-*^ embryos compared to wild-type siblings. (K-L) The profiles of *tfap2a* and *tfap2c* at 24 hpf again remain similar to the two previous time points across all genetic combinations. (M) At 24 hpf the levels of *foxd3* are not significantly different across any genotypes. (N) The levels of *sox10* are markedly down in only the *tfap2a*^*-/-*^;*tfap2c*^*-/-*^ embryos and levels of *sox9b* are unchanged across all genotypes (O). Statistical significance of below 0.05 adj p-value is denoted with a *. Not all significant differences have been labelled. NS is to emphasise pairwise comparisons which fail an adj. p-value <0.05 cut-off.

Our next question was how the transcript levels of *tfap2a* and *tfap2c*, along with three well characterised neural crest-specific genes (*foxd3*, *sox10* and *sox9b*), behaved across all nine genotypes and the three developmental stages (Fig 3A–3O). At 4 somites, embryos homozygous for either *tfap2a* or *tfap2c* had significantly lower transcript abundances for their respective genes, indicating that nonsense-mediated decay [56] had most likely occurred (Fig 3A and 3B). A genetic interaction is evident in *tfap2a*^*-/-*^*;tfap2c*^*+/+*^ embryos between *tfap2a* and *tfap2c* with higher levels of wild-type *tfap2c* transcripts than in wild-type siblings (Fig 3B) while *tfap2a* is not increased in the inverse case of *tfap2a*^*+/+*^;*tfap2c*^*-/-*^ mutants (Fig 3A). This indicates that, by the 4 somite stage, the neural crest GRN is able to detect reduced levels of *tfap2a* in knockouts and compensation through upregulation of *tfap2c* is established. Interestingly, at 4 somites and partially at 15 somites, in homozygous *tfap2a* mutants transcript levels of the three NC genes follow the *tfap2c* expression pattern. For example, among embryos homozygous for *tfap2a*, *sox9b* expression at 4 and 15 somites is highest in *tfap2a*^*-/-*^*;tfap2c*^*+/+*^ embryos, but drops to half in *tfap2a*^*-/-*^*;tfap2c*^*+/-*^ embryos (Fig 3E and 3J). This suggests a direct quantitative relationship between *tfap2* transcript abundance and that of its targets.

By 24 hpf the abundance of *tfap2a* and *tfap2c* across the nine genotypes remains much the same as at the previous developmental stages (Fig 3K and 3L). Interestingly, *foxd3* and *sox9b* levels are no longer significantly different in *tfap2a*^*-/-*^*;tfap2c*^*-/-*^ embryos, which is suggestive of their exit from the neural crest GRN or initiation of expression in non-neural crest tissues, but levels of *sox10* remain strongly reduced in the double mutants (Fig 3M–3O). Also, *tfap2a*^*-/-*^*;tfap2c*^*+/-*^ embryos now have levels of *foxd3*, *sox9b* and *sox10* comparable to wild type which suggests a general recovery of the neural crest GRN by this stage. These data show that the time point of the strongest molecular phenotype and *tfap2c* compensation is at around 4–15 somites with the morphological phenotypes beginning to emerge by 15 somites.

### RNA-seq on *tfap2a;tfap2c* knockouts at 15 somites confirms 3’ tag sequencing data and produces a more detailed transcriptional landscape

To further investigate the dose-dependent compensation while also creating a more detailed transcriptomic profile of pluripotent and differentiating neural crest cells, we carried out RNA-seq on *tfap2a;tfap2c* knockouts at the 15 somite stage. All 9 genotypes were assessed using a total of 90 single embryos. Principal component analysis highlights that *tfap2a*^*-/-*^*;tfap2c*^*-/-*^ and *tfap2a*^*-/-*^*;tfap2c*^*+/-*^ are most similar on a molecular level in spite of their vastly different morphological phenotypes (S2A Fig). Pairwise comparisons of four different genotypes to their wild-type siblings shows high numbers of genes changing in both *tfap2a*^*-/-*^*;tfap2c*^*-/-*^ and *tfap2a*^*-/-*^*;tfap2c*^*+/-*^ groups (S2B Fig, Table 1). The majority of significant genes have reduced transcript levels in double mutants with robust p-values (S2C Fig). The 15 somite 3’ tag sequencing and RNA-Seq data sets showed good correlation of the detected DE genes at an adjusted p-value < 0.01 (S2D Fig)

Hierarchical clustering on the significantly changed genes from the *tfap2a*^*-/-*^*;tfap2c*^*-/-*^ versus wild type pairwise comparison and ZFA enrichment placed genes into functional groups. While loss of both *tfap2a* and *tfap2c* leads to a reduction in genes involved in neural crest and epidermis development it also leads to an upregulation of genes associated with neural terms (S2E Fig).

### Identifying genes required for *tfap2a;tfap2c* knockout compensation / neural crest rescue

The 3’ tag sequencing analysis had highlighted that both *tfap2a*^*-/-*^*;tfap2c*^*-/-*^and *tfap2a*^*-/-*^*;tfap2c*^*+/-*^ gave the most extensive molecular phenotypes even though *tfap2a*^*-/-*^*;tfap2c*^*+/-*^ were morphologically indistinguishable from wild-type siblings at 15 somites whereas *tfap2a*^*-/-*^*;tfap2c*^*-/-*^ presented obvious morphological phenotypes by that stage. Hence a single wild-type allele of *tfap2c* is sufficient to rescue the morphological *tfap2a*^*-/-*^*;tfap2c*^*-/-*^ neural crest specification and differentiation phenotype despite the observed effect on the transcriptional level. We were therefore keen to understand which genes are involved and may be required for the rescue of the morphological phenotype.

**Fig 4:**
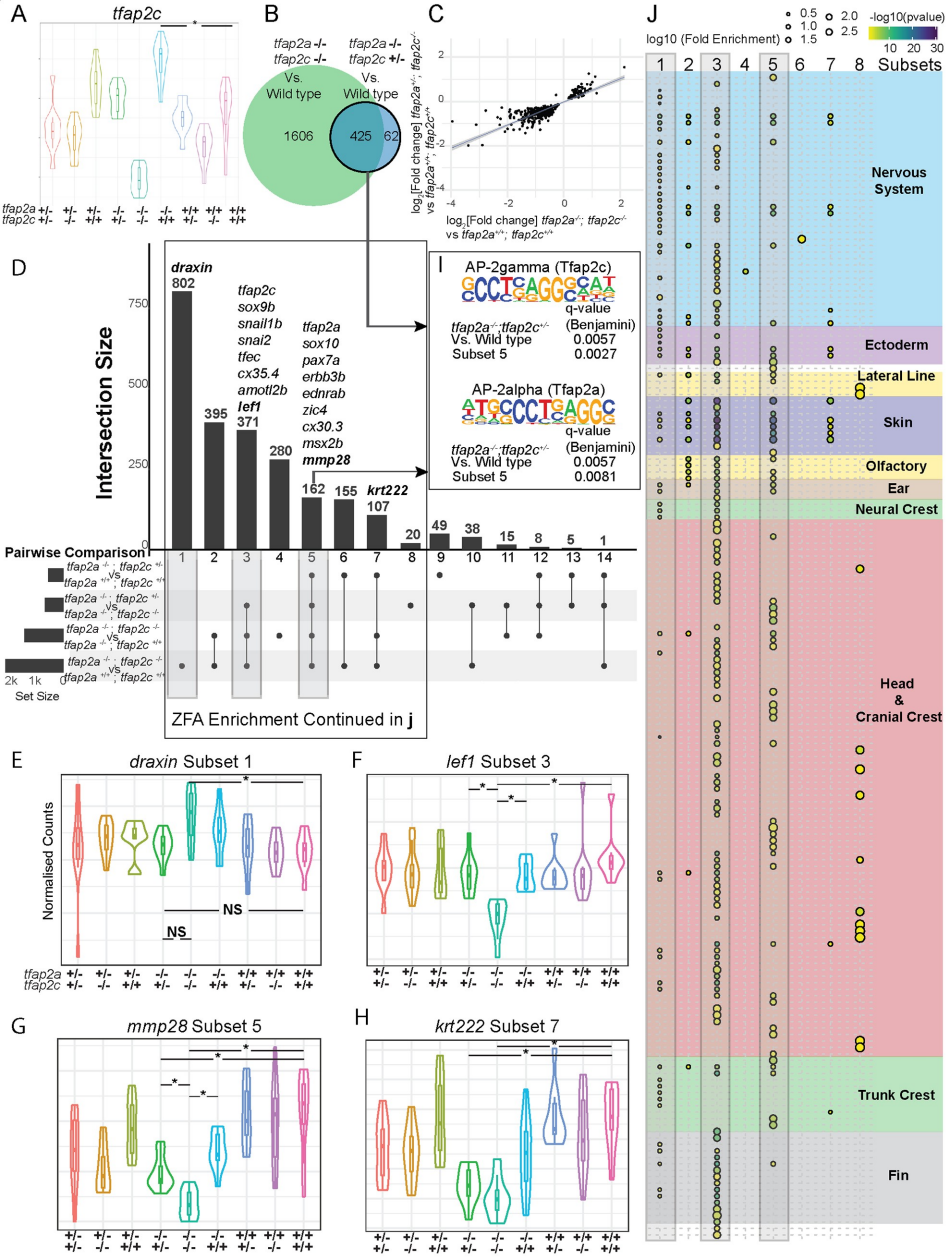
Identification of NC-specific gene subsets in *tfap2a;tfap2c* mutant RNA-seq 15 somite data. (A) RNA-seq at 15 somites, an * indicates a significant (adj. p-value <0.05) increase of *tfap2c* transcript in *tfap2a*^*-/-*^;*tfap2c*^*+/+*^ embryos when compared to wild-type siblings. (B) Overlapping gene lists comparison of significantly (adj. p-value <0.05) differentially expressed genes when *tfap2a*^*-/-*^;*tfap2c*^*-/-*^ and *tfap2a*^*-/-*^;*tfap2c*^*+/*-^ are compared to wild-type siblings. (C) *tfap2a*^*-/-*^;*tfap2c*^*+/*-^ log_2_[fold change] plotted against *tfap2a*^*-/-*^;*tfap2c*^*-/-*^ log_2_[fold change] with regression curve showing a 1:2 ratio. (D) Subsetting of gene lists from four different pairwise comparisons. The subsets are labelled 1–14 and the genes from (e-h) are noted at the top of the groups they belong to. Groups 1, 3 and 5 have grey boxes around them. (E-H) Examples of violin plots for the four subset groups with “*” signifying a <0.05 adj p-value between two groups and NS indicating not significant. Genotypes of the embryo groups are listed at the bottom of each plot. (I) Enrichment for AP-2alpha (Tfap2a) and AP-2gamma (Tfap2c) binding sites in *tfap2a*^*-/-*^;*tfap2c*^*+/*-^ DGE list and subset 5. (J) ZFA enrichment was carried out on all 14 subsets but only returned significant enrichment for groups 1–8. The log_10_[Fold Enrichment] is designated by the size of the circle and the colour represents -log_10_[p-value]. Grey bars correspond to the same subsets in (D). Anatomy terms have been manually organised based on the themes to the right. The actual terms have been cropped and placed in (S3 Fig ZFA Enrichment) for ease of reading.

First, we assessed expression of *tfap2c* in the 15 somites RNA-seq data and found that the levels of *tfap2c* were significantly higher in *tfap2a*^-/-^ embryos when compared to wild-type embryos (Fig 4A) demonstrating active regulatory compensation rather than redundancy. We then compared the sets of DE genes derived from the pairwise comparisons of wild type with *tfap2a*^*-/-*^*;tfap2c*^*-/-*^ and *tfap2a*^*-/-*^*;tfap2c*^*+/-*^. The vast majority of DE genes in the *tfap2a*^*-/-*^*;tfap2c*^*+/-*^ condition were also changed in the *tfap2a*^*-/-*^*;tfap2c*^*-/-*^ embryos (Fig 4B). Crucially, this set is enriched for genes with a dose-dependent response to successive loss of *tfap2a/c* alleles where for each gene the log_2_ fold change in *tfap2a*^*-/-*^*;tfap2c*^*+/-*^ is about half that in *tfap2a*^*-/-*^*;tfap2c*^*-/-*^ (Fig 4C). This demonstrates that loss of a third *tfap2a/c* allele affects the neural crest GRN, however the transcriptional changes are not sufficient to derail neural crest specification and differentiation. Together this identifies a core set of *tfap2a/tfap2c*-responding genes, separate from secondary downstream events caused by differentiation failure and tissue loss.

As a single wild-type allele of *tfap2c* is able to maintain neural crest specification we sought to identify genes that are sensitive to different levels of *tfap2c* by dissecting the full ablation response (*tfap2a*^*-/-*^*;tfap2c*^*-/-*^) using the partial ablation profiles (*tfap2a*^*-/-*^*;tfap2c*^*+/+*^ *and tfap2a*^*-/-*^*;tfap2c*^*+/-*^). To this end we ran four differential gene expression (DGE) analyses: double homozygous embryos against embryos with one or two wild-type alleles of *tfap2c*, and wild-type embryos against *tfap2a*^*-/-*^*;tfap2c*^*-/-*^ or *tfap2a*^*-/-*^*;tfap2c*^*+/-*^. Next we overlapped the four lists to produce 14 subsets (Fig 4D). This identified groups of genes that share distinct expression profiles. Subset one contains genes where *tfap2a*^*-/-*^*;tfap2c*^*-/-*^ knockout resulted in a mild, but significant, change from wild-type siblings but there is no significant difference between *tfap2a*^*-/-*^*;tfapc*^*+/-*^ and *tfap2a*^*-/-*^*;tfap2c*^*-/-*^ or wild-type siblings (Fig 4E). For genes in subset three a complete *tfap2a*^*-/-*^*;tfap2c*^*-/-*^ knockout resulted in a significant change from wild-type siblings while a single allele of *tfap2c* was sufficient to return the expression to wild-type levels. An example of this case would be *lef1* (Fig 4F). Subset five contained genes that are only partially rescued, for example *sox10*, *pax7a*, *ednrab*, *kctd15a* and *erbb3b*, all of which play vital roles in neural crest development across vertebrates [37]. A single wild-type allele of *tfap2c*, or even both wild-type alleles, is unable to return expression to wild-type levels but the expression is still significantly different from the *tfap2a*^*-/-*^*;tfap2c*^*-/-*^ condition, as exemplified by *mmp28* (Fig 4G). Finally, subset seven contained genes that are only rescued by two alleles of *tfap2c*, such as *krt222* (Fig 4H).

We next analysed the gene sets for transcription factor motif enrichment using HOMER[57] (Fig 4I, see Table 1 for figshare link to all significant results). The full DGE list for *tfap2a*^*-/-*^*;tfap2c*^*-/-*^ against wild type (Fig 4B) had 31 enriched known motifs (q-value < 0.05), which included Ap2gamma and Ap2alpha. These motifs also appeared in the top three of 13 enriched motifs for the DGE list for *tfap2a*^*-/-*^*;tfap2c*^*+/-*^ against wild type, suggesting that this profile reflects a core set of Ap2 targets. Analysis of the subsets in Fig 4D revealed that only subset five, which contains genes showing dosage sensitivity and partial rescue in *tfap2a*^*-/-*^*;tfap2c*^*+/-*^ embryos, had an enrichment for Ap2 targets. Subset three, the set of genes fully rescued by one or two wild-type copies of *tfap2c*, was enriched mostly for binding sites of zinc-finger domain-containing transcription factors such as KLFs, but also Tead2, a transcription factor involved in Yap/Taz Hippo signalling[58,59].

### ZFA enrichment confirms specific neural crest signatures

For a functional gene analysis, we carried out ZFA enrichment on all 14 gene subset lists which yielded significant enrichments for subsets 1–8 (Fig 4J, S3 Fig). Subset three, the genes fully rescued by either one or two alleles of *tfap2c*, showed the strongest enrichment for terms associated with the neural crest, head and cranial crest and also fin. While fin enrichment may seem nonsensical for a 15 somite embryo, this is due to the fact that many genes annotated for fin development are also involved in craniofacial development. A similar enrichment profile resulted from subset five, the genes where either one or two alleles of *tfap2c* rescued expression levels to a significant extent, but not completely. By contrast, the two largest subsets, containing genes that change in double homozygous embryos with respect to wild types, but not compared to *tfap2a*^*-/-*^*;tfapc*^*+/-*^, showed a bias towards nervous system and ectoderm enrichment. Crucially, subsets six and seven with genes that failed to be rescued by either one (subset seven) or two (subset six) wild-type *tfap2c* alleles, had no or very little neural crest enrichment. This suggests these genes represent *tfap2a* targets outside of neural crest differentiation.

Taken together the enrichment analysis breaks down the full *tfap2a/tfap2c* knockout response into separate expression classes with different functional profiles. Subsets three and five contain genes that are fully or partially rescued by *tfap2c*, show the strongest neural crest enrichment and are thus most likely to represent the core of the *tfap2* neural crest GRN. Interestingly, only the partial rescue gene list (subset five) is enriched for direct Ap2 targets, suggesting that the full rescue list (subset three), which shows the strongest neural crest signature, contains more genes that are further downstream in the network.

### Markov clustering reveals *tfap2a* and *tfap2c-*specific gene clusters

**Fig 5:**
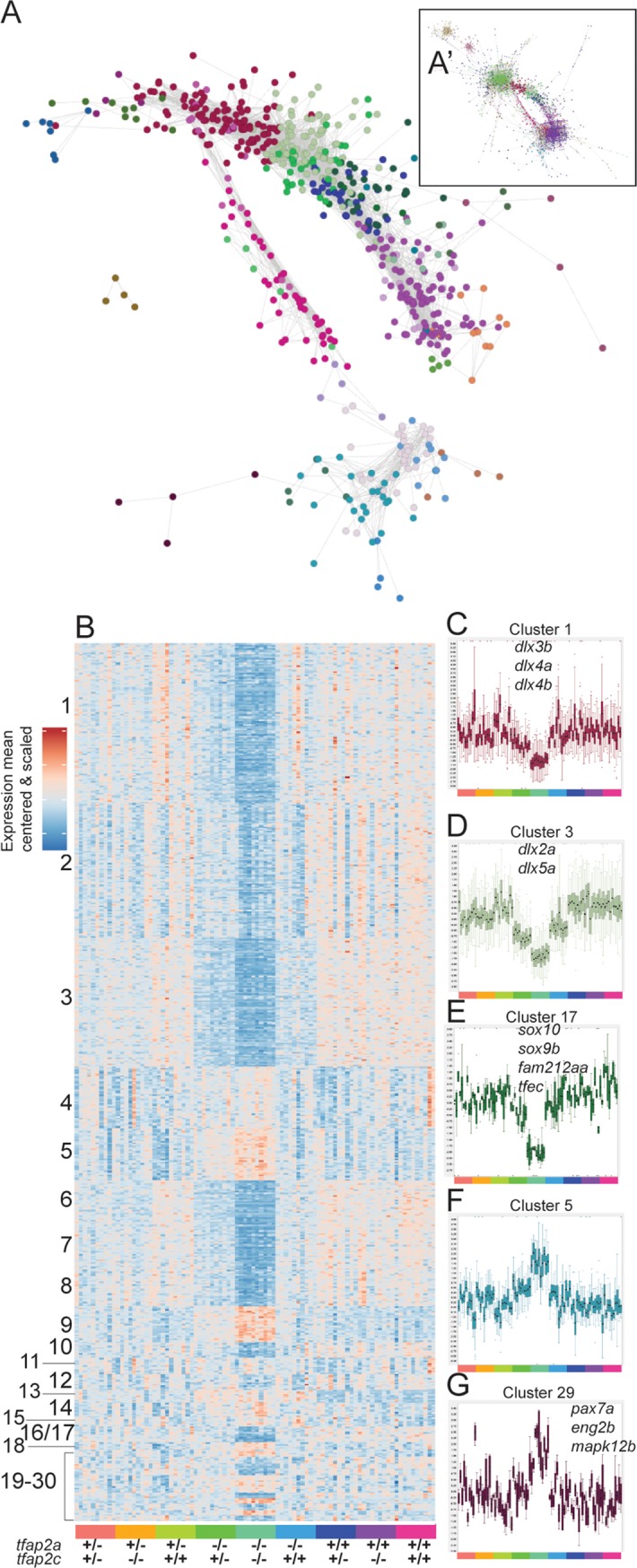
Network analysis and Markov clustering of RNA-seq 15 somite data set. (A) Interaction network analysis of 15 somite (0.7 Pearson correlation) RNA-seq data set represented as a subset. The entire interaction network can be found in A’. Each node represents a single gene and its colour corresponds to its cluster group. (B) A mean-centred and scaled heatmap representing 30 MCL network clusters organised by cluster size and by genotype at the bottom. (C-G) Examples of individual clusters displayed as boxplots of the values for all the genes in the cluster (mean centered and variance scaled). Samples are arranged as in (B) and are colour coded at the bottom of each cluster. Cluster number corresponds to the same cluster in (B). Some genes contained in clusters are labelled on the plot.

Next we applied an expression correlation network and Markov clustering approach using Biolayout *Express*^*3D*^ [60,61] to identify co-expression profiles independent from condition-driven differential expression analysis. We constructed a network graph with genes as nodes and their Pearson correlation as edges from the *tfap2a;tfap2c* 15 somite RNA-seq dataset and used Markov clustering (MCL) to divide the network into discrete sets of co-expressed genes. The network clusters isolated co-expression groups of genes that share a genomic locus (Fig 5A’ and S4 Fig) similar to our observation in the *sox10* and *mitfa* mutants, but also identified *tfap2a* and *tfap2c*-specific components (Fig 5A) within the larger co-expression network.

In total 30 clusters containing a total of 600 genes were driven by changes in the *tfap2a* or *tfap2c* genotypes (Fig 5B). It is important to point out that in the previous analysis we compared lists derived from pairwise DGE comparisons, whereas MCL clusters genes based on their expression similarity across all samples. Therefore, these clusters might exclude genes that are identified in the DESeq2 analysis because of low expression correlation with other genes, but also include highly correlated genes which did not produce a significant adjusted p-value in the DESeq2 analysis.

The unsupervised clustering confirmed the strong signal in the double homozygous fish (clusters one and two) and dose-dependent compensation by *tfap2c* (cluster three). However, in addition it provided increased functional resolution. For example, cluster 17 (Fig 5E) was highly specific to neural crest effectors containing the *soxE* paralogues *sox10* and *sox9b*, the micropthalmia bHLH transcription factor *tfec*, as well as the Pak4 kinase inhibitor *fam212aa*, and one uncharacterised gene (*si*:*ch211-243g18*.*2; ENSDARG00000044261*).

The differentiation of the neural crest also requires the down-regulation of specific groups of genes, for example to repress a neural fate. Cluster five (Fig 5F) contains a collection of *soxB* family genes (*sox3*, *sox19a*, *sox19b*, *sox21b*), one of which (*sox19)* is one of the earliest CNS markers in vertebrates [47]. Cluster five also includes another example of paralogues of *oct-*related transcription factors *pou3f2b* (*Oct-2*) and *pou3f3a*, which are associated with controlling CNS development. Cluster 29 (Fig 5G) contains a collection of genes (*pax7a*, *eng2b*, *mapk12b* and *enfa2a*) which, based on the midbrain/hindbrain expression patterns of *pax7a* and *eng2b*, also suggests a developmental CNS role. All gene lists of individual clusters, along with GO and ZFA enrichments, can be found in (Table 1).

Using many replicates of single, genotyped embryos from the same clutch has allowed us to show how a single allele of *tfap2c* is sufficient to maintain a minimal neural crest GRN. Based on this we have compiled functional subsets of maintained genes, many of which are still poorly described and previously have never been associated with the neural crest. We have identified multiple cases where gene families or paralogues behave in the same manner, highlighting more potential examples of the compensatory nature of the GRN in general. To validate the association of novel genes with neural crest biology, we next analysed a set of candidates using a knockout approach.

### Validation of novel neural crest transcripts

Our transcriptional profiles from mutants and FACS enriched neural crest cells contain a large number of novel neural crest candidate genes with poor or no functional annotation (Table 1). We chose a subset of these for further analysis based on lack of functional annotation and their differential expression across the different datasets (Table 2). To validate their involvement in neural crest biology, we analysed the expression patterns or screened for knockout phenotypes in zebrafish embryos. For example, transcripts of the gene *wu*:*fc46h12; ENSDARG00000114516* were strongly reduced in several *sox10* mutant experiments (Table 1). At 24 hpf *wu*:*fc46h12* has an identical expression pattern to the xanthophore marker *gch2* in wild-type and *sox10* mutants (S5A, S5D, S5G and S5H Fig), but diverges at 48 hpf in wild types as *wu*:*fc46h12’s* expression domain becomes more specific to a ventral crest population (S5E and S5F Fig), heart and dorsal aorta (S5E’ and S5F’ Fig). The majority of these expression domains are also lost at 48 hpf in *sox10* mutants (S5G and S5H Fig). A CRISPR/Cas9 knockout allele, *wu*:*fc46h12*^*sa30572*^, was homozygous viable. Maternal-zygotic (MZ) mutant *wu*:*fc46h12*^*sa30572*^ embryos from intercrosses of homozygous females with heterozygous males showed heart oedema at 36 hpf (S5I and S5J Fig). By 5 dpf most larvae formed swim bladders and had grossly normal hearts. A more detailed analysis will be required to ascertain the role of *wu*:*fc46h12* in heart development.

**Table 2:** Knockouts investigated in this study.

Gene	Allele	Gene_ID	DGE Experiment	Phenotype	Notes
*add3b*	*sa10508*	ENSDARG00000056250	FACS	NP	Hom-Viable
*akr1b1*	*sa30578*	ENSDARG00000006215	*sox10 & tfap2a;tfap2c*	PT	
*anxa2a*	*sa24716*	ENSDARG00000003216	FACS	NP	
*c10orf11*	*sa12051*	ENSDARG00000057166	FACS	NP	Hom-Viable
*cax1*	*sa10712*	ENSDARG00000045601	*sox10 & tfap2a;tfap2c*	PT	Hom-Viable MZ-PT
*defbl1*	*sa30573*	ENSDARG00000075161	*sox10*	NP	
*Hipk2*	*sa23245*	ENSDARG00000042518	FACS	NP	
*itga6a*	*sa21422*	ENSDARG00000042282	tfap2a;tfap2c	NP	
*itga6b*	*sa16239*	ENSDARG00000069946	tfap2a;tfap2c	NP	
*si*:*ch211-155k24*.*6*	*sa13099*	ENSDARG00000062688	*sox10*	NP	
*si*:*ch211-155k24*.*6*	*sa13185*	ENSDARG00000062688	*sox10*	NP	
*slc27a4*	*sa21677*	ENSDARG00000017047	FACS	NP	
*smarca4*	*sa12514*	ENSDARG00000077226	*tfap2a;tfap2c*	PT	
*tspan36*	*sa21320*	ENSDARG00000024540	*sox10*	NP	
*wu*:*fc46h12*	*sa30572*	ENSDARG00000104814	*sox10 & yap1*	PT	Hom-Viable
*yap1*	*sa25458*	ENSDARG00000068401	*tfap2a;tfap2c*	PT	Hom-Viable MZ-PT
*yap1*	*sa25474*	ENSDARG00000068401	*tfap2a;tfap2c*	PT	Hom-Viable MZ-PT

Two genes, *akr1b1* and *cax1*, were differentially expressed in the *tfap2a;tfap2c* and *sox10* mutant data sets (Table 1). The former, *akr1b1*, is ubiquitously expressed [47] and, using CRISPR/Cas9, we created a premature stop, *akr1b1*^*sa30579*^. Homozygous *akr1b1*^*sa30579*^ fish develop normally but presented pale xanthophores (S5K Fig).

A premature stop in *cax1* was already available from the Zebrafish Mutation Project. A previous report has shown expression in xanthophores and upon morpholino knockdown a reduction in neural crest tissues of the jaw as well as xanthophores [62]. While zygotic *cax1*^*sa10712*^ homozygotes present a dulling in the colouring of xanthophores (S5L Fig) and rounding up of the normally highly dendritic cells, they lack obvious craniofacial phenotypes. Homozygous *cax1*^*sa10712*^ adults are viable and fertile, but MZ*cax1*^*sa10712*^ embryos appear ventralised at somitogenesis pointing to a role for *cax1* during early embryonic development (S5M Fig).

### A role for maternally supplied *yap1* in melanocytes

**Fig 6:**
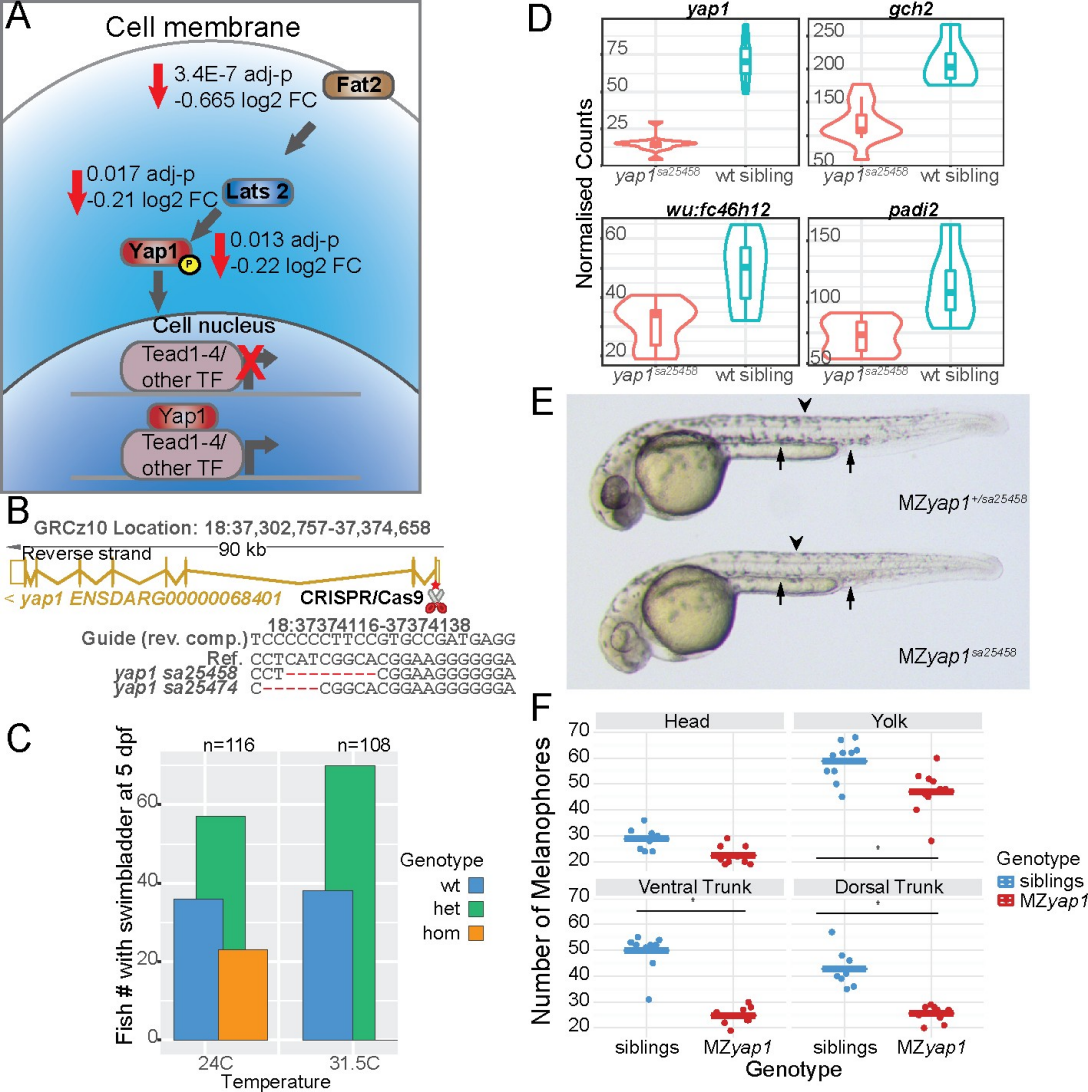
*yap1* mutants are temperature sensitive and *yap1* plays a role in melanocyte development. (A) Transcripts of members of the Hippo signalling pathway *fat2*, *lats2* and *yap1* were less abundant in *tfap2a;tfap2c* mutants when compared to wild-type siblings. A schematic showing their role in signal transduction and transcription inside a cell. (B) CRISPR/Cas9 mutations were made in the first exon of *yap1* leading to the two alleles described. The exon-intron structure of the *yap1* transcript is shown in gold. The exact deletions are displayed below. (C) Embryos from a single clutch were split and raised at 24C and 31.5C. Bars indicate the absolute number of fish forming a swim bladder at 5 dpf for each *yap1*^*sa25458*^ genotype (D) Normalised counts of 3’ tag sequencing data at 24 hpf comparing *yap1*^*sa25458*^ mutants to wild-type siblings. All four genes, *yap1*, *gch2*, *wu*:*fc46h12* and *padi2*, have an adj. p-value <0.05. (E) Maternal zygotic *yap1* mutants present a strong reduction in melanocyte numbers at 36 hpf at both dorsal (arrow head) and ventral tail regions (arrow). (F) Quantification of melanocytes with the quantities on the left and then broken down into the regions of the head, yolk, ventral tail and dorsal tail. Each dot represents a region in a single larva, siblings in blue and MZ*yap1*^*sa25458*^ in red. A statistical significance of <0.05 is indicated with “*”.

Expression of the transcriptional regulator *yap1* was reduced in double homozygous embryos in our 4 somite *tfap2a;tfap2c* 3’ tag sequencing data (Table 1) and *yap1* was also enriched in neural crest FACS-sorted cells (Fig 2E). We also found that three members of the Hippo signalling pathway, *fat2*, *lats2* and *yap1*, had significant negative log_2_ fold changes in the 15 somite *tfap2a;tfap2c* knockout versus wild type RNA-seq dataset (Fig 6A). Furthermore, subset three from the *tfap2a/tfap2c* analysis was enriched for genes with a Tead2 binding site, an interactor of Yap/Taz transcription factors. These data suggest a very early role for Hippo signalling in neural crest cells. Previous work has identified Hippo signalling as a coactivator of Pax3, involved in melanocyte gene expression [63], and *in vitro* studies suggest that YAP is involved in neural crest fate and migration [64].

### *yap1* knockouts are temperature sensitive, homozygous viable and reduced in body size

To investigate the role of *yap1* in neural crest we targeted its first exon using CRISPR/Cas9 and created two alleles, *yap1*^*sa25458*^ and *yap1*^*sa25474*^, leading to frame shifts and premature stops (Fig 6B). When heterozygous carriers for either *yap1*^*sa25458*^ or *yap1*^*sa25474*^ were intercrossed and embryos raised at 28.5°C we found the previously described ocular phenotypes at 48–72 hpf in approximately 25% of embryos [65], albeit with variable penetrance depending on incubator temperature. We therefore tested whether these two *yap1* mutants were temperature sensitive by splitting a single clutch and raising the embryos at two different temperatures. When raised at 24°C, by 5 dpf just under a quarter (22 of 116) of larvae with normal morphology and a filled swim bladder were homozygous *yap1* mutant. By contrast, when raised at 31.5°C none of the larvae with a swim bladder (108) were homozygous mutant for *yap1* leaving a 1:2 ratio of 38 wild-type and 70 heterozygous fish (Fig 6C).

We raised intercrosses of *yap1* carriers for each allele (*yap1*^*sa25458*^ & *yap1*^*sa25474*^) at a permissive temperature of 24°C until 5 dpf then transferred them to our standard fish nursery to test for adult viability of homozygotes. At two months post fertilisation, a subset of these fish seemed smaller than their siblings (S6A Fig). We measured and genotyped intercrosses from both *yap1* alleles and confirmed that *yap1* homozygous fish were viable, but smaller than their wild-type siblings (S6B Fig).

### Zygotic *yap1* mutants show signs of neural crest GRN disruption

Although zygotic *yap1* mutants did not display obvious morphological phenotypes in neural crest cell types, we investigated potential neural crest GRN changes. We intercrossed *yap1*^*+/sa25458*^ carriers, raised them at 28.5°C and collected embryos for 3’ tag sequencing at 4 somites, 15 somites and 24 hpf. The transcriptome profiles were normal at 4 somite and 15 somite stages, with the majority of the changed genes on the same chromosome as *yap1* (Fig 1J, Table 1). However, at 24 hpf the early xanthophore pigment cell marker *gch2* and *wu*:*fc46h12*, the newly identified pigment marker described above, were significantly reduced in *yap1* mutants (Fig 6D). Interestingly, the early epidermis marker *padi2* was also reduced (Fig 6D).

### Loss of maternal *yap1* mRNA causes reduced melanocyte numbers at 30 hpf

Previous studies have shown a role for *yap1* in very early development of zebrafish and medaka [66–68]. In zebrafish, this precedes zygotic genome activation and thus highlights a role for maternally deposited transcripts. The developmental time course data of *yap1* expression confirmed high levels of maternally deposited polyadenylated *yap1* (E-ERAD-475, www.ebi.ac.uk/gxa/home/).

Given the maternal deposition of *yap1* transcripts in the egg, we crossed heterozygous male *yap1*^*+/sa25458*^ carriers to homozygous female *yap1*^*sa25458*^ fish and evaluated the resulting MZ*yap1*^*sa25458*^ larvae at the restrictive temperature of 31.5C. At approximately 30 hpf we observed a strong reduction in the number of melanocytes in roughly half of the embryos. The previously described ocular phenotype [65] was also apparent in addition to a mild pericardial oedema (Fig 6E). These larvae are otherwise morphologically stage matched. To quantify the melanocyte reduction we counted melanocytes in four different sections (head, yolk, ventral trunk and dorsal trunk) of each larva and then genotyped them. A significant melanocyte reduction of about 50% in the yolk, ventral tail and dorsal tail was found, with no major difference in the number of melanocytes in the head (Fig 6F). This demonstrates that maternally deposited mRNA is able to rescue a melanocyte phenotype at 30 hpf further highlighting the very early induction of the neural crest GRN.

## Discussion

We have used transcriptional profiling of mutants affecting different steps of neural crest specification and differentiation to dissect the zebrafish neural crest GRN. We have used 3’ tag sequencing as a first pass screening method to then hone in with more detailed RNA-seq. To make our data easily accessible to the research community we have made the *tfap2a*;*tfap2c* 15 somite RNA-seq experiment available for browsing and downloading in Expression Atlas (https://www.ebi.ac.uk/gxa/experiments/E-MTAB-6106/Results). The analysis of genotyped single embryos, independent from a visible phenotype, has allowed us to separate transcriptional responses from morphological outcomes. This approach is complementary to cell type-specific assays which require tissue manipulation and/or dissociation, much like the neural crest FACS RNA-seq data set described here. It is important to keep in mind that RNA-seq measures absolute differences in transcript abundance, rather than up- and down-regulation of genes. In whole embryos these abundances can be changed by altered tissue size as well as a change in mRNA levels in an otherwise unaffected tissue. Recently, elegant methods have been developed to biotag specific cells *in vivo* and isolate their nuclei for further processing [69]. However, currently, these require the pooling of embryos which would be challenging to apply to non-phenotypic embryos in loss of function analyses.

### Initiation of neural crest GRN expression before gastrulation, shortly after zygotic genome activation

The neural crest is typically described as being induced at the lateral edges of the neural plate after gastrulation. However, using wild-type developmental time course data we can place the activation of the neural crest transcription factors *tfap2a*, *tfap2c* and *foxd3* at the Dome stage, which follows zygotic genome activation and precedes gastrulation. In zebrafish, simultaneous loss of *tfap2a* and *foxd3* has been shown to genetically ablate the neural crest [70,71] with *tfap2a* and *foxd3* expressed in mutually exclusive compartments of the embryo at the shield stage, midway through gastrulation. The overlap of these expression domains forms the presumptive neural crest [71]. In *Xenopus laevis* a high degree of overlap exists in the blastula pluripotent GRN and the neural crest GRN with the neural crest retaining the pluripotency of cells in the blastula stage rather than being induced later on in development [72]. Interestingly, the activation of the neural crest marker *crestin* also coincides with the Dome stage (E-ERAD-475, www.ebi.ac.uk/gxa/home/). This suggests that the establishment of the proto-neural crest GRN comes shortly after zygotic genome activation and places its initiation much earlier than previously shown in zebrafish and other vertebrates. This also raises the possibility of maternal mRNAs playing a larger role than previously thought in early neural crest initiation. Nevertheless we do not dispute that the neural crest cell lineage is likely to pass through a well-defined ectodermal state as elegantly shown in single cell studies [73].

### Genetic ablation of the neural crest

In addition to *tfap2a;foxd3* loss of function, a combined knockout of *tfap2a* and *tfap2c* genetically ablates the neural crest in zebrafish [17,71]. In the case of *tfap2a;foxd3*, *tfap2a* is thought to have an activator function whereas *foxd3* has been shown to act both as a repressor and an activator [5]. Knockouts of *tfap2a* fail to form normal jaws and have reduced numbers of melanocytes, but still form neural crest cells. On a transcriptional level, using 3’ tag sequencing, the number of genes which are differentially abundant in the *tfap2a* or *tfap2c* mutants alone are modest, 39 and 5 genes respectively at the 15 somite stage (Fig 2E). At the 4 somite stage *tfap2c* acts in a compensatory manner as its overall abundance is increased by almost 50% in *tfap2a*^-/-^ embryos (Fig 3B and Fig 5A). By removing a single wild-type *tfap2c* allele in *tfap2a*^*-/-*^ embryos the number of changing genes jumps from 39 to 1152 (Fig 2E), although this extensive change in gene expression is marked morphologically by only a mild decrease in the numbers of melanocytes in the tail at a much later stage. Using RNA-seq at the 15 somite stage increases the total numbers of changing genes detected but the general trends remain much the same. *tfap2* family proteins are thought to form both homodimers as well as heterodimers [74]. This stepwise genetic ablation implies that *tfap2c* does not require *tfap2a* to initiate the early neural crest GRN and that either homodimers of *tfap2c* alone or potentially heterodimers with other *tfap2* family members are sufficient; however, we do not see upregulation of any other *tfap2* genes.

### Dissection of the neural crest transcriptional network

Previous work in mouse, frog, chick and fish have identified the important role of AP-2 to initiate the neural crest and drive its downstream targets such as *msx1*, *sox9/10* and *snail*. [12,75,35,16]. Most previous analysis of AP-2 downstream targets have taken a gene by gene approach or, in the case of ChIP-seq, return thousands of putative targets. With our large-scale screen our intent is to identify the bulk of relevant AP-2 downstream targets and to make these available for further analysis. For example, *Msx1* is a well-defined AP-2 target and our analysis shows transcripts for both paralogues, *msx1a* and *msx1b*, are less abundant in the *tfap2a/c* double knockouts, suggesting that a double knockout is necessary to abolish *msx1* function. A further aspect is that AP-2 has been shown to play an important role in epidermis in addition to neural crest. Both of these tissues arise at similar time points from ectoderm, and it is therefore crucial to separate the neural crest from the ectoderm signal. By combining multiple mutant data sets over developmental time along with the neural crest FACS data set we could establish the timing of when different levels of the neural crest GRN begin. Along with a large number of known downstream targets the subsets contain many uncharacterised genes, suggesting a role for these in pigmentation. We can further group genes which are more likely to not be specific to the neural crest but rather involved in epidermis development (Fig 2F). Using the overlaps across the three different time points we have classified groups of genes from an “early” role to “mid” and then “later.” We appreciate that both *tfap2a* and *tfap2c* have broad expression domains and our attempts to separate out neural crest and epidermal-specific signals will have limitations. Due to the shared lineage of neural crest and dorsal epidermis cells, it is also possible that GRN overlap exists before the lineage splits. Single cell studies will be better able to address these issues. We have also further characterised trunk neural crest and melanocyte-specific downstream targets by analysing *sox10* and *mitfa* knockouts. Similar approaches could be used in the future to address different neural crest sub lineages, for example, in *sox9b* mutants.

### Neural crest identity requires repression of a neural fate

The 15 somite stage had the highest number of differentially expressed genes in the *tfap2a*;*tfap2c* loss of function model and therefore we chose to investigate this stage in more detail using RNA-seq. Using different subsetting approaches we have characterised distinct groups of neural crest genes and also have identified the core neural crest GRN that is maintained via *tfap2c*. The hierarchical clustered heatmap (S2E Fig) highlights an enrichment of neural genes that are increased in the mutant samples. Considering the emerging model that neural crest cells are not actually induced *in situ* but rather a refinement of pluripotent blastula cells [72], our data support the notion that not only is the activation of the neural crest GRN important but also the repression of non-neural crest-specific GRNs.

### Compensation of *tfap2a* knockout phenotypes via *tfap2c* and identification of genes involved in the neural crest rescue

Both our 3’ tag sequencing time course and RNA-seq reveal a great disparity between the severity of molecular phenotypes and morphological phenotypes. This data set allows us to identify the genes required to maintain neural crest induction but also what levels of expression are sufficient. RNA-seq analysis of *tfap2a*;*tfap2c* knockouts and their siblings revealed an increase of *tfap2c* mRNA expression in *tfap2a* mutants at 15 somites. Although not addressed in this study, an interesting question now is: what is the molecular machinery which identifies the need for genetic compensation and how is it carried out? We find that whereas a single allele of *tfap2c* is able to rescue the early morphological neural crest ablation phenotype the expression of a core set of downstream effectors cannot be restored to wild-type levels. This separates the morphological phenotype, and its secondary molecular effects, from the primary gene regulatory effect of *tfap2* loss of function. We have used this differential behaviour of downstream targets to identify genes which *tfap2c* is able to return to wild-type levels or to only partially rescue from the *tfap2a/c* double knockout. This has confirmed known neural crest players but has also added new genes to the neural crest GRN. The genes in subsets three and five (Fig 4C–4H) represent a core set of 371 and 162 genes, respectively, of the neural crest GRN required for early neural crest initiation and are most likely to be of high developmental and evolutionary importance. An enrichment for AP-2 transcription factor binding sites in the partially rescued gene subset is consistent with the first tier nature of the genes.

### Genetic compensation via paralogues

Humans are particularly susceptible to haploinsufficient mutations in a number of neural crest-specific genes, including *sox10*, leading to Waardenburg syndrome or Hirschsprung disease, whereas this is less the case in zebrafish [76]. *sox10*^*+/-*^ fish are adult viable and are phenotypically normal. Based on the developmental timing and clustering behaviour of the *soxE* family paralogues *sox10* and *sox9b*, there is a good probability that these two genes are able to compensate for each other in early neural crest cells. Similarly, fish with mutations in *tfap2c* are homozygous viable and *tfap2a*^*+/-*^*;tfap2c*^*-/-*^ fish are indistinguishable from their wild-type siblings. By contrast, alterations of *TFAP2A*, acting in a dominant negative manner, lead to a number of developmental phenotypes in humans.

Phenotype-driven forward genetics screens [77,78] are very successful in identifying mutations affecting a multitude of processes across the zebrafish lifespan. By contrast, reverse genetics screens have demonstrated, against expectations, that many presumably protein-disrupting mutations fail to lead to an obvious morphological phenotype in the first five days zebrafish of development [39,79]. Although more sensitive screening assays across different life stages and conditions are required to identify more subtle phenotypes, a multi-gene loss of function approach may be required to counteract as of yet poorly characterised mechanisms of compensation. Here, using the neural crest as a model, we dissect the relationship between transcriptional robustness and morphological outcomes. Our study has also begun to reveal more evidence of genetic compensation in other paralogous genes. Unsupervised clustering has highlighted that entire gene families clustered together across development [42] and behaved in a similar manner in different genetic combinations in the *tfap2a*;*tfap2c* loss of function experiments (Fig 5B–5E, Table 1).

Another example of possible paralogous compensation can be observed in the relatively mild developmental phenotypes of the *yap1* knockouts. Recently double knockouts of *yap1* and *taz (wwtr1)*, its paralogue, have shown much stronger early developmental phenotypes and are embryonic lethal [67]. A deeper understanding of genetic and functional paralogues with respect to mutual compensation versus division of function will provide mechanistic insight into gene function evolution.

### A role for Hippo signalling in the neural crest

We have identified a reduction in the abundance of some Hippo signalling members in both our 3’ tag sequencing and RNA-seq data sets. Previously, a role for Hippo signalling has been suggested in the neural crest using conditional mouse knockout models and in cell culture [80,63,64]. However, in the case of the mouse, complete *yap1* knockouts are not viable and in human iPS neural crest cell models both *YAP1* and *TAZ* (*WWTR*) require modulation. Yap1 itself is not capable of binding DNA but requires TEAD elements also identified in our studies (Table 1 gene lists). A role for TEADs in both melanocytes and melanoma has been previously documented [81] and placing *yap1* downstream of Ap2 signalling adds an interesting aspect to Hippo signalling in melanocytes. In zebrafish we show a role for maternally deposited *yap1* in the differentiation of melanocytes, however the effect on other neural crest subtypes remains to be investigated. It is also likely that the *yap1* paralogue *taz* could be playing a compensatory role. Furthermore, transcriptional analysis of non-phenotypic zygotic mutant embryos raised at permissive temperatures shows dis-regulation of several neural crest and epidermis genes. This is a further example of a transcriptional phenotype in the absence of morphological changes. Over the past few years post-embryonic neural crest stem cells have been identified in mouse and zebrafish [26,82,83]. The temperature sensitive *yap1* signalling model described here allows for the conditional inactivation of Hippo signalling and could also be combined with *taz* heterozygous fish. This would allow for the investigation of Hippo-dependent processes in post-embryonic neural crest stem cells, but also growth, pattern formation and regeneration later in development and in adults.

### Conclusions

Taken together, we have used transcriptional profiling and stepwise genetic ablation of the neural crest to divide the neural crest GRN into temporal and functional units containing new candidate genes alongside well known factors. The analysis of paralogue compensation separates the morphological neural crest ablation phenotype from the first expression changes to the core *tfap2* GRN. We confirm association of previously uncharacterised genes through knockout experiments and demonstrate a role for maternal transcripts in pigment cell development. Future studies of the functional gene clusters described here will help to further refine their role in neural crest development as well as their involvement in human genetic disorders and diseases such as neuroblastoma and melanoma.

## Materials and methods

### Ethics statement

Zebrafish were maintained in accordance with UK Home Office regulations, UK Animals (Scientific Procedures) Act 1986, under project licences 80/2192, 70/7606 and P597E5E82. All animal work was reviewed by The Wellcome Trust Sanger Institute Ethical Review Committee.

### Zebrafish husbandry and phenotyping of mutants

Zebrafish were maintained at 23.5°C on a 14 h light/10 h dark cycle. Male and female zebrafish from genotyped heterozygous fish carrying mutations were separated overnight before letting them spawn naturally the next day. Fertilised eggs were grown at 28°C and single or multi-allelic phenotyping was carried out as previously described [39,84]. The *sox10*^*t3*^ and *sox10*^*baz1*^ alleles were a gift from Robert Kelsh and *mitfa*^*w2*^ was previously a gift from Jim Lister [25,34].

### Embryo collection

Embryos were either morphologically sorted into phenotypically abnormal and normal (*sox10*^*t3/baz1*^ collected at 28 hpf, 36 hpf and 48 hpf) or collected blind at the stage of interest. Single embryos were placed individually into the wells of a 2 ml deep well block (Axygen, Cat number P-DW-20-C-S), snap frozen on dry ice and then stored at -80°C.

### FACS

22–23 hpf embryos were collected from the zebrafish transgenic *sox10*:*mg* line which labels neural crest nuclei with mCherry and crest cell membranes with GFP. We observed a delay in the membrane-bound GFP signal causing two separate neural crest populations; one labelled only with the nuclear mCherry marker, and a second labelled both with mCherry and the membrane-bound GFP (Fig 1H). We sorted these two populations separately along with a third non-transgenic population for pairwise differential expression analysis, however for the purposes of this study we pooled the neural crest cell data. We also generated transcriptional profiles of cranial crest and trunk crest separately to capture lowly expressed genes specific to those cell types. We therefore separated heads and tails of embryos from the same stage and isolated individual cranial and trunk neural crest populations from each tissue comprising both mCherry+ and mCherry+/GFP+ cells as well as an unlabelled non-crest population. All cell populations were processed to produce polyA RNA-seq libraries and sequenced. Dissociated cells from about 30–50 larvae were collected for FACS as previously described (Manoli et al., 2012). Briefly, embryos were dechorionated using 33 mg/ml pronase (Sigma) and pooled either as whole embryos or as pools of heads and tails. The yolks were removed using deyolking buffer (55 mM NaCl, 1.8 mM KCl, 1.25 mM NaHCO_3_) followed by digestion with trypsin-EDTA. Finally, the pellet was resuspended in FACSmax Cell Dissociation solution (AMS Biotechnology) and dissociated cells collected by passing the suspension through a 20 μm cell strainer (Sysmex Partec). Using appropriate gating, dissociated cells were sorted into mCherry positive, mCherry and GFP positive and unlabelled non-crest cells on the BD INFLUX. The data was analysed using FlowJo.

Sorted cells were collected and lysed in 110 μl of RLT buffer (Qiagen) containing 1 μl of 14.3M beta mercaptoethanol (Sigma). The lysate was allowed to bind to 1.8 volumes of Agencourt RNAClean XP (Beckman Coulter) beads for 10 min and RNA was eluted from the beads as per the manufacturer’s instructions. Total RNA was converted into cDNA libraries using the SMART-Seq V4 Ultra Low Input RNA kit (Clontech) followed by Nextera DNA Library Prep kit (Illumina) as per manufacturer’s instructions. Libraries were pooled and sequenced on Illumina HiSeq 2000 in 75 bp paired-end mode.

### Nucleic acid extraction

Frozen embryos were lysed in 100 μl RLT buffer (Qiagen) containing 1 μl of 14.3M beta mercaptoethanol (Sigma). The lysate was allowed to bind to 1.8 volumes of Agencourt RNAClean XP (Beckman Coulter) beads for 10 min. The plate was then applied to a plate magnet (Invitrogen) until the solution cleared and the supernatant was removed without disturbing the beads. While still on the magnet the beads were washed three times with 70% ethanol and RNA was eluted from the beads as per the manufacturer’s instructions. RNA was quantified using either Qubit RNA HS assay or Quant-iT RNA assay (Invitrogen).

### Genotype confirmation

Genotyping was carried out as described previously [40]. Briefly, 1 μl of DNA from the extracted total nucleic acid was used to confirm the genotype of each sample using KASP SNP and InDel identification assays (LGC group) designed against our allele of interest. The genotyped plates were read on a plate reader (Pherastar, BMG Labtech) and 10–12 samples per genotype were selected for making libraries.

### Transcript counting

DeTCT libraries were generated as described previously [50]. Briefly, 300 ng of RNA from each genotyped sample were DNAse treated, fragmented and bound to streptavidin beads. The 3’ ends of the fragmented RNA were pulled down using a biotinylated polyT primer. An RNA oligo containing the partial Illumina adapter 2 was ligated to the 5’ end of the bound fragment. The RNA fragment was eluted and reverse transcribed using an anchored oligo dT reverse transcriptase primer containing one of the 96 unique index sequences and part of the Illumina adapter 1. The Illumina adapters were completed during a library amplification step and the libraries were quantified using either the BioPhotometer (Eppendorf) or Pherastar (BMG Labtech). This was followed by size selection for an insert size of 70–270 bases. Equal quantities of libraries for each experiment were pooled, quantified by qPCR and sequenced on either HiSeq 2000 or HiSeq 2500.

Sequencing data were analysed as described previously [50]. Briefly, sequencing reads were processed with the DeTCT detag_fastq.pl script and aligned to the GRCz10 reference genome with BWA 0.5.10. The resulting BAM files were processed using the DeTCT pipeline, which results in a list of regions representing 3’ ends, together with a count for each sample. These counts were used for differential expression analysis with DESeq2 on pairwise combinations of samples. Each region was associated with Ensembl 86 gene annotation based on the nearest transcript in the appropriate orientation. False positive 3’ ends, representing, for example, polyA-rich regions of the genome, were filtered using the DeTCT filter_output.pl script with the—strict option, reducing the number of 3’ ends from 439,367 to 53943. Gene sets were analysed using topgo-wrapper for GO enrichment and Ontologizer for ZFA enrichment.

### RNA-seq

Total nucleic acid was isolated from *tfap2a*^*+/sa24445*^*;tfap2c*^*+/sa18857*^ intercrosses at 15 somites. KASP genotyping was used to identify all nine possible genotypes. Total nucleic acid was treated with DNAseI (NEB, Catalogue number M0303L) and 10 replicates per genotype were processed. Ambion ERCC spike-in mix 2 (Cat. No. 4456740) was added to 200 ng RNA according to the manufacturer’s instructions and sequencing libraries were prepared using the Illumina TruSeq Stranded mRNA Sample Prep Kit. Libraries were pooled and sequenced on Illumina HiSeq 2500 in 75 bp paired-end mode.

Sequencing data were assessed using FastQC and aligned to the GRCz10 reference genome and Ensembl 86 transcriptome using TopHat2. Read counts per gene were generated using htseq-count and used as input for pairwise differential expression analysis with DESeq2. Gene sets were analysed using topgo-wrapper for GO enrichment and Ontologizer for ZFA enrichment. Custom R scripts were used for hierarchical clustering and principal component analysis.

### Transcription factor motif analysis

Transcription factor motif enrichment was performed using HOMER's findMotifs.pl tool (v4.10.3) with default settings. The GRCz10 promoter set used was created with HOMER's updatePromoters.pl tool based on RefSeq genes from -2000 bp to 2000 bp relative to the TSS.

### Graph based Pearson correlation and Markov clustering of RNA-Seq data

Count data were clustered using Biolayout *Express*^*3D*^. Graph based network visualization with a Pearson correlation of above 0.7 and Markov clustering was carried out using Biolayout *Express*^*3D*^ (https://www.ebi.ac.uk/about/news/service-news/BioLayoutExpress3D). Markov clusters were visually inspected and extracted for display as a heatmap using the geneExpr (https://github.com/richysix/geneExpr) Shiny App (Fig 5).

### Embryo and fin clip genotyping

Genotyping of embryos and fin clips was performed as previously described [39,40]. A schematic of all genes with the positions of their respected mutations can be found in S6 Fig. Previously unpublished alleles used in this study are listed in Table 3.

**Table 3:** New alleles used in this study.

Allele name	Pos(Assembly:Chr:Pos)	WT	MUT
*cax1*^*sa10712*^	GRCz11:4:14984215	T	A
*tfap2c*^*sa18857*^	GRCz11:6:56171775	G	T
*tfap2a*^*sa24445*^	GRCz11:24:8725695	T	A
*yap1*^*sa25474*^	GRCz11:18:37355128	AT	A
*yap1*^*sa25458*^	GRCz11:18:37355126	TCATCGGCA	T
*wu*:*fc46h12*^*sa30572*^	GRCz11:2:7638034	ATCAGGGTGAAGGTCAGCAGCAAT	A
*akr1b1*^*sa30578*^	GRCz11:4:14901038	GTCCGGCTACCGGCACA	G

### Zebrafish anatomy enrichment

Zebrafish anatomy enrichment is a similar approach to the widely used Gene Ontology enrichment but instead uses zebrafish anatomical terms linked to zebrafish genes. The enrichment is performed using ontologizer (http://ontologizer.de/) with the ontology from http://ontologies.berkeleybop.org/zfa.obo. ZFIN gene IDs are linked to ZFA terms using http://zfin.org/downloads/phenoGeneCleanData_fish.txt and http://zfin.org/downloads/wildtype-expression_fish.txt and Ensembl IDs are converted to ZFIN IDs using Ensembl. Ontologizer is then run with the Parent-Child-Union calculation and Bonferroni multiple testing correction.

### RNA whole mount *in situ* hybridisation

RNA DIG-labelled probes were generated from cDNA libraries (Transcriptor High Fidelity cDNA Synthesis Kit, Roche) covering all relevant embryonic stages. PCR was performed and then TA cloned using TOPO-TA (Invitrogen). RNA riboprobes were produced using the T7- and SP6-promoter sequence, enabling *in vitro* transcription of the plasmid using T7- and SP6-RNA polymerase (Roche). All oligonucleotide sequences are listed here:

wu:fc46h12_left1:CTGCTGACCTTCACCCTGATTCTG, wu:fc46h12_right1:GGTGTATTGCCTAAAACCCTCAGC wu:fc46h12_left2:ATTGCTGCTGACCTTCACCCTGAT, wu:fc46h12_right2:ATTGCCTAAAACCCTCAGCTTCCA.

### CRISPR/Cas9

Creation and identification of CRISPR/Cas9 zebrafish alleles were conducted as previously described using the zebrafish codon optimised double NLS Cas9 [85,41].

## Supporting information

S1 Fig Zebrafish anatomy enrichment of *sox10* and *mitfa* mutant profiles across time. ZFA enrichment was tested for all *sox10* and *mitfa* mutants compared to wild-type siblings at all time points shown in Fig 1J but only time points at 24 hpf or later returned significantly enriched terms (adj. p-value <0.05).
(TIF)

S2 Fig RNA-seq transcriptomic analysis of *tfap2a;tfap2c* mutants at 15 somite stage. (A) Principal component analysis of replicates of all 9 *tfap2a;tfap2c* genotypes showing the first two principal components. Dots represent a single embryo and genotype is denoted by colour. (B) Bars denote the numbers of genes for four most relevant pairwise combinations (adj. p-value <0.05) with the numbers of genes with a positive log_2_ fold change in green (above the line) and negative in red (below the line). The specific genotypes of *tfap2a* and *tfap2c* are listed across the top for each bar. (C) A pairwise comparison of RNA-seq of *tfap2a*^*-/-*^;*tfap2c*^*-/-*^ versus their wild-type siblings at 15 somites. The adj p-value is on the y-axis and the log_2_ fold change on the x-axis. (D) Comparison of 3’ tag sequencing (y-axis) and RNA-seq (x-axis) log_2_ fold change of genes with an adj p-value <0.01 in the *tfap2a*^*-/-*^;*tfap2c*^*-/-*^ versus wild-type siblings pairwise comparison showing an overall linear correlation. (E) Heatmap of gene expression with an adj p-value <0.05 from *tfap2a*^*-/-*^;*tfap2c*^*-/-*^ to wild-type siblings pairwise comparison. Genes are hierarchically clustered with the samples organised by genotype. ZFA enrichment analysis was carried out on clusters as indicated by the black boxes. ZFA enrichments with their corresponding significances are depicted on the right. ZFA terms were further broadly categorised into epidermis, neural crest and neuronal.
(TIF)

S3 Fig Enlargement of zebrafish anatomy enrichment from Fig 4H. (TIF)

S4 Fig Examples of haplotype-specific signals from the *tfap2a*;*tfap2c* 15 somite RNA-seq data set. (A, D, F) Markov clusters from BioLayout *Express*^*3D*^ expression analysis shown in Fig 5 A’ which contain genes linked to a specific region on a particular chromosome. A bar indicating the genotypes of the embryos is at the bottom. (A) A cluster of genes located on chromosome 24 linked to *tfap2a*. Genes behave in three groups depending on whether *tfap2a* is heterozygous, homozygous or wild type. A recombination has occurred in one embryo (circled in red) in the *tfap2a* homozygous group and that cluster of genes now behaves as the wild-type condition. (B) A list of genes and their chromosomal positions which make up the cluster in (A). (C) A karyotype map of chromosome 24 showing the location of *tfap2a* (blue arrow head right) and the positions of the genes contained in the cluster (red arrow heads left). (D) A cluster of genes on chromosome 15 where genes fall into two different groups, indicating one of the parents would have been heterozygous for the region. (E) A list of the genes contained in the cluster which are mostly on chromosome 15 and potentially two incorrectly mapped genes. (F-G) A third example of a haplotype-specific region located on chromosome 16 where both parents are presumably heterozygous for the region leading to three different groups.
(TIF)

S5 Fig Functional analysis of pigment cell-specific genes. (A-H) Whole mount *in situ* analysis of *wu*:*fc46h12* and *gch2* as a pigment cell comparison. *sox10*^*baz/+*^ heterozygotes embryos as sibling controls (A-B) and mutant *sox10*^*baz1*^ embryos at 24 hpf (C-D). At 48 hpf *in situs* were carried out on *albino* embryos to serve as wild-type controls (E-F) with arrows indicating the heart and arrow heads the dorsal aorta. A blow up of this region can be found in E’-F’. (G-H) *E*xpression of *gch2* and *wu*:*fc46h12* at 48 hpf in *sox10*^*baz1*^ mutants. I-J Wild-type and MZ*wu*:*fc46h12*^*sa30587*^ embryos at 36 hpf with oedema around the forming heart (J). (K) Wild-type sibling and mutant *akr1b1*^*sa30579*^ at 4 dpf with mutant larvae presenting a reduction of yellow colour produced by xanthophores. Magnifications indicated with a black box. (L) Wild-type sibling and mutant *cax1*^*sa10712*^ larvae at 5 dpf. Close ups indicated by black boxes around the head show dull yellow colour and abnormal cell morphology in mutants (arrow head). (M) MZ*cax1*^*sa10712*^ phenotype at 19 somite stage.
(TIF)

S6 Fig Adult *yap1* mutant fish are smaller than wild-type siblings. (A) Homozygous *yap1* mutants are viable but present with a variation in size. (B) Quantification of size at two months of age with the corresponding genotypes for both *yap1* alleles. A statistically significant difference with p-val <0.05 is indicated by “*”.
(TIF)

S7 Fig Structure of genes and position of mutations used in this study. Filled boxes represent coding sequence, unfilled ones denote untranslated regions.
(TIF)

S1 Table Complete list of ZFA enrichments from Fig 2F. (TSV)

S2 Table List of 26 genes from Fig 2F group 13. (TXT)

S3 Table Metadata IDs for all sequencing data. (TXT)
