# Supplementary figures and images for: The gene regulatory basis of genetic compensation during neural crest induction
Source: PLoS Genet. 2019 Jun 14;15(6):e1008213. doi: 10.1371/journal.pgen.1008213 (PMC6594659; doi:10.1371/journal.pgen.1008213)

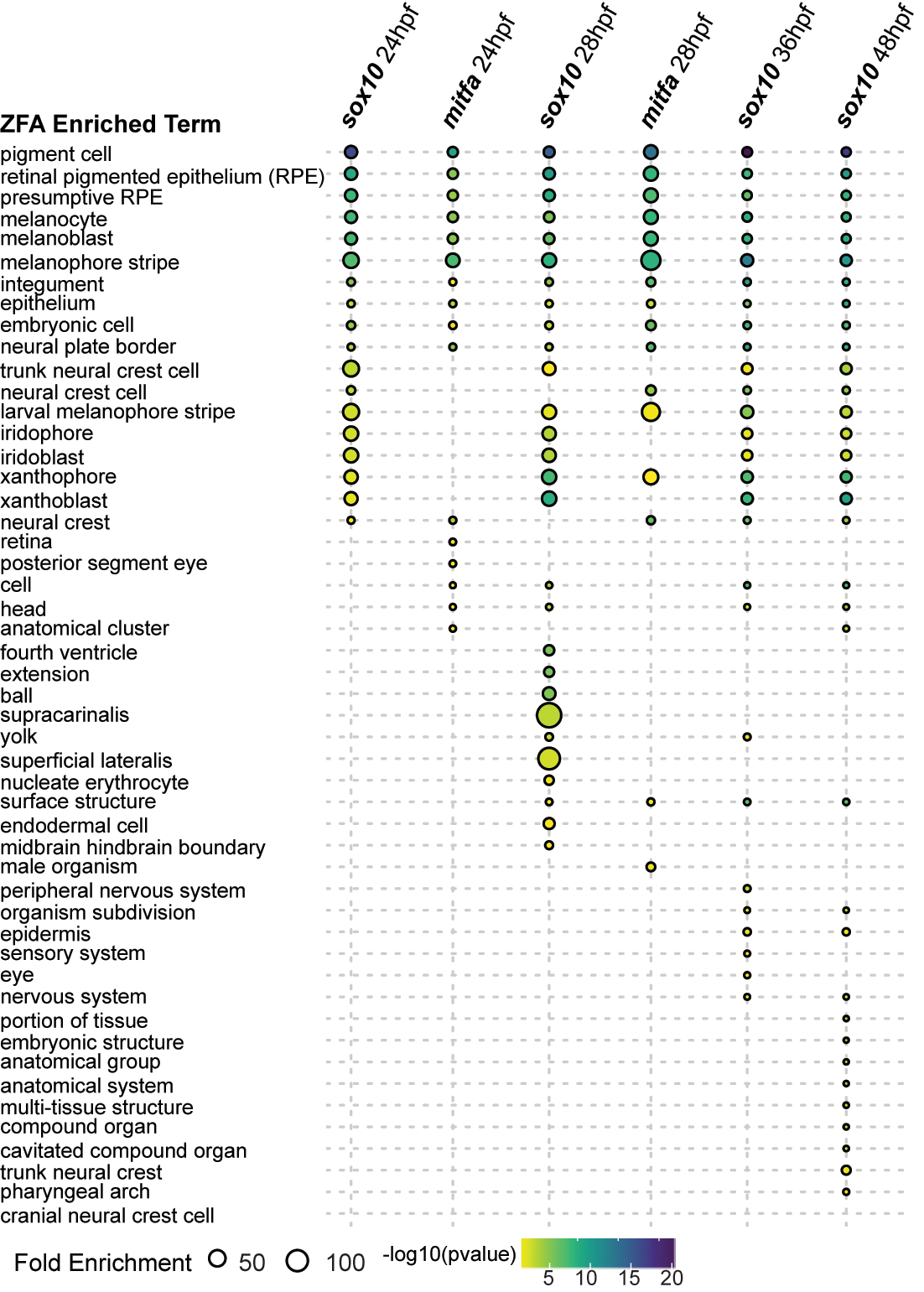

Supplement: S1 Fig — ZFA enrichment was tested for all sox10 and mitfa mutants compared to wild-type siblings at all time points shown in Fig 1J but only time points at 24 hpf or later returned significantly enriched terms (adj. p-value <0.05). (TIF) [file pgen.1008213.s001.tif]

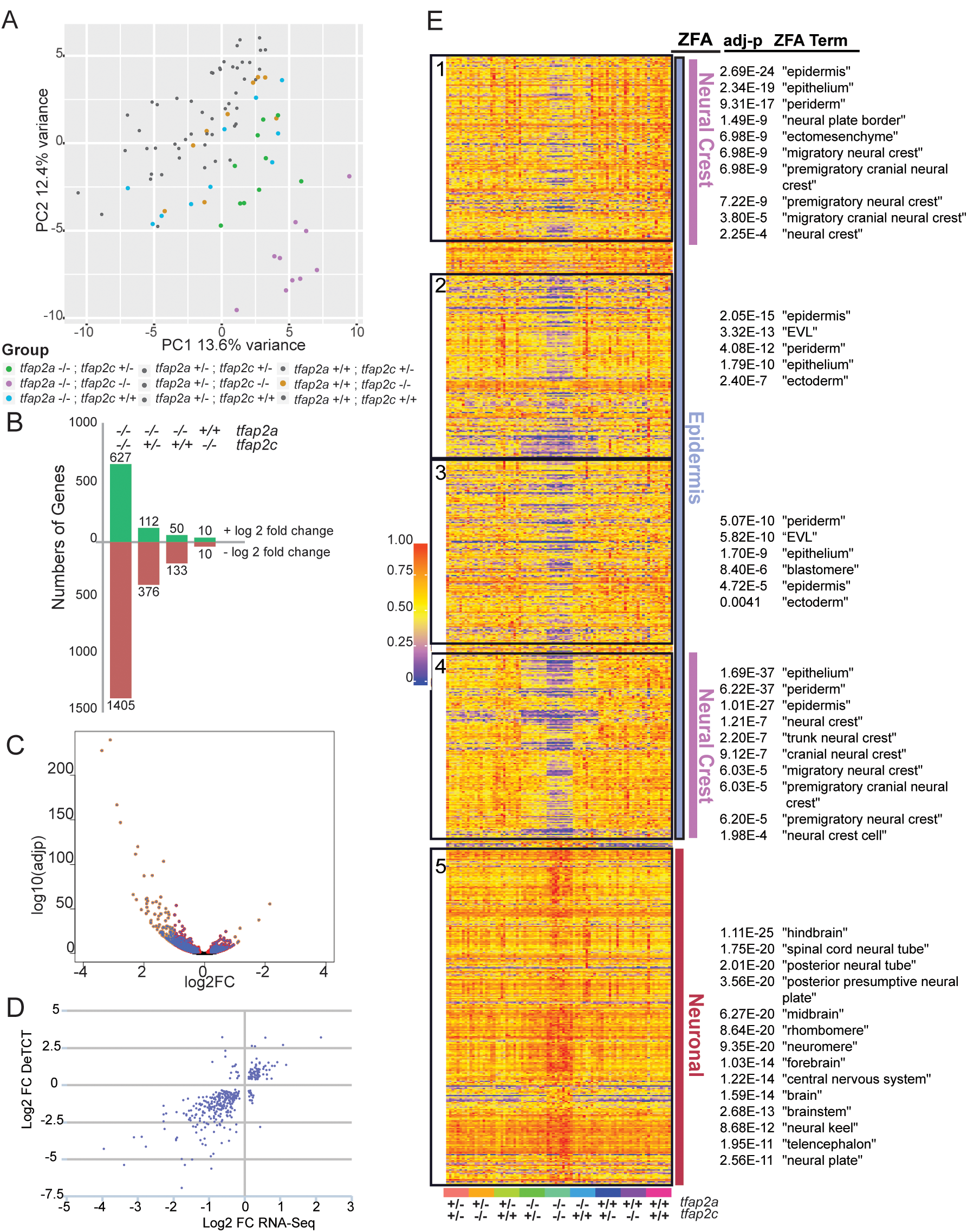

Supplement: S2 Fig — (A) Principal component analysis of replicates of all 9 tfap2a;tfap2c genotypes showing the first two principal components. Dots represent a single embryo and genotype is denoted by colour. (B) Bars denote the numbers of genes for four most relevant pairwise combinations (adj. p-value <0.05) with the numbers of genes with a positive log2 fold change in green (above the line) and negative in red (below the line). The specific genotypes of tfap2a and tfap2c are listed across the top for each bar. (C) A pairwise comparison of RNA-seq of tfap2a-/-;tfap2c-/- versus their wild-type siblings at 15 somites. The adj p-value is on the y-axis and the log2 fold change on the x-axis. (D) Comparison of 3’ tag sequencing (y-axis) and RNA-seq (x-axis) log2 fold change of genes with an adj p-value <0.01 in the tfap2a-/-;tfap2c-/- versus wild-type siblings pairwise comparison showing an overall linear correlation. (E) Heatmap of gene expression with an adj p-value <0.05 from tfap2a-/-;tfap2c-/- to wild-type siblings pairwise comparison. Genes are hierarchically clustered with the samples organised by genotype. ZFA enrichment analysis was carried out on clusters as indicated by the black boxes. ZFA enrichments with their corresponding significances are depicted on the right. ZFA terms were further broadly categorised into epidermis, neural crest and neuronal. (TIF) [file pgen.1008213.s002.tif]

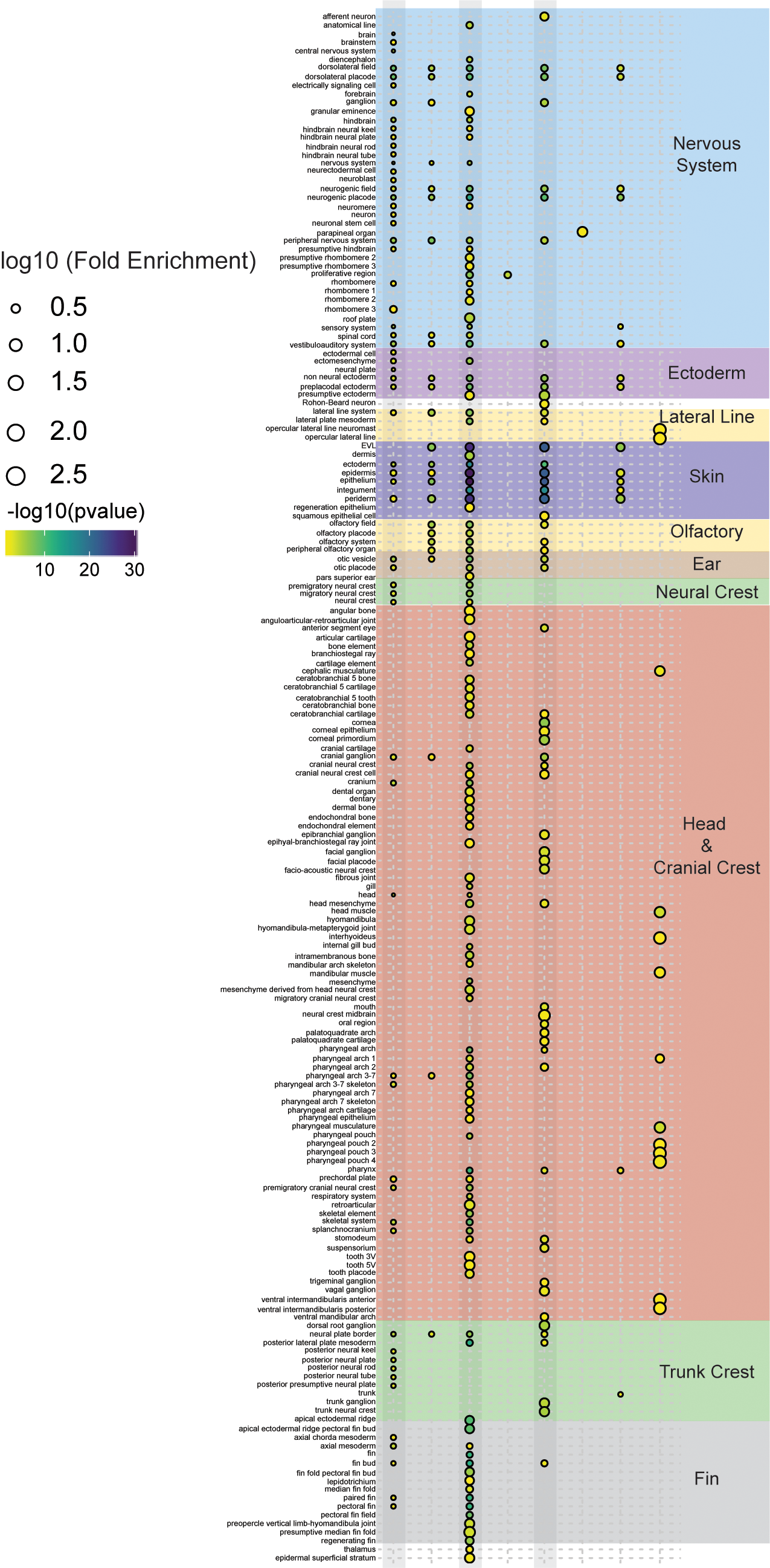

Supplement: S3 Fig — (TIF) [file pgen.1008213.s003.tif]

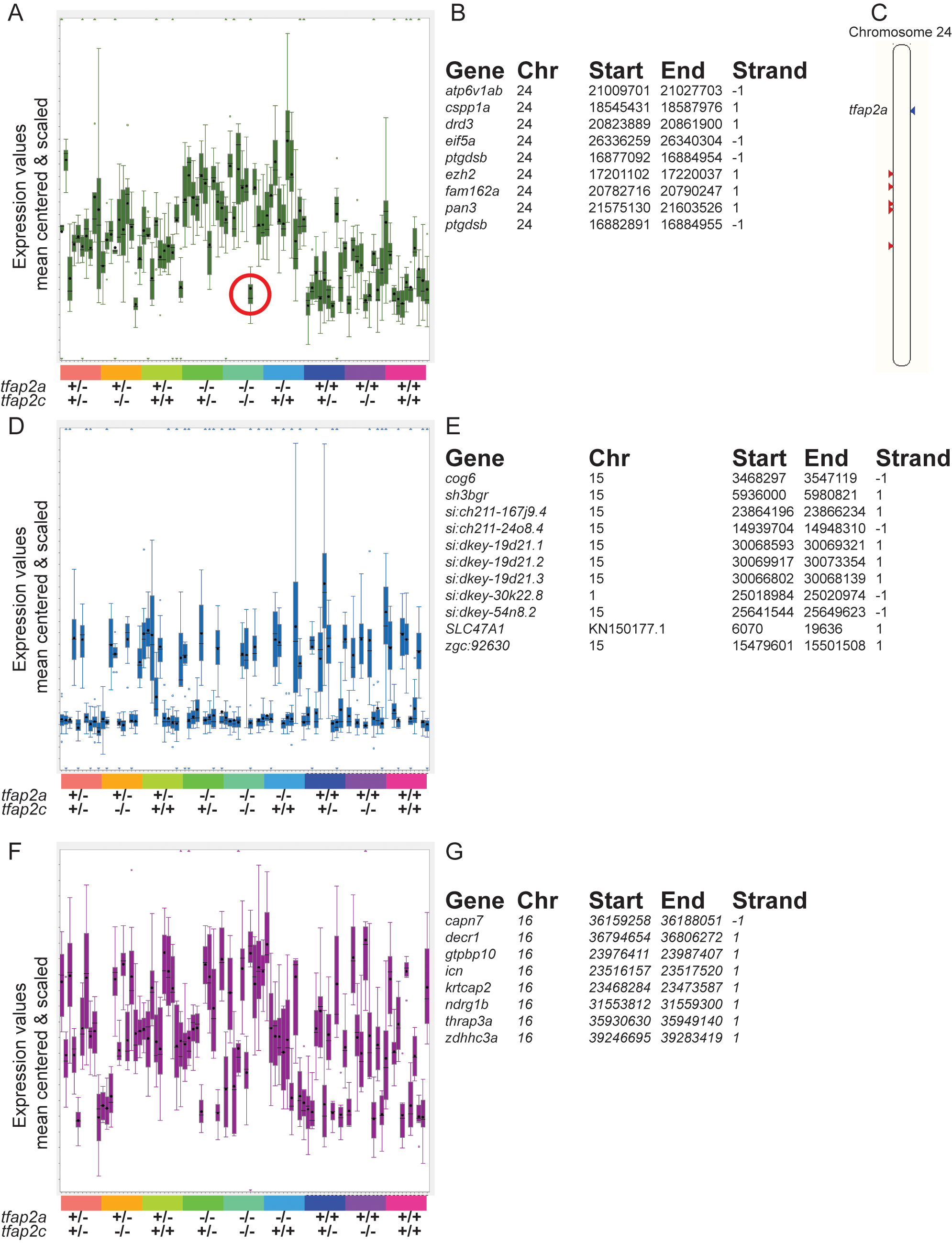

Supplement: S4 Fig — (A, D, F) Markov clusters from BioLayout Express3D expression analysis shown in Fig 5 A’ which contain genes linked to a specific region on a particular chromosome. A bar indicating the genotypes of the embryos is at the bottom. (A) A cluster of genes located on chromosome 24 linked to tfap2a. Genes behave in three groups depending on whether tfap2a is heterozygous, homozygous or wild type. A recombination has occurred in one embryo (circled in red) in the tfap2a homozygous group and that cluster of genes now behaves as the wild-type condition. (B) A list of genes and their chromosomal positions which make up the cluster in (A). (C) A karyotype map of chromosome 24 showing the location of tfap2a (blue arrow head right) and the positions of the genes contained in the cluster (red arrow heads left). (D) A cluster of genes on chromosome 15 where genes fall into two different groups, indicating one of the parents would have been heterozygous for the region. (E) A list of the genes contained in the cluster which are mostly on chromosome 15 and potentially two incorrectly mapped genes. (F-G) A third example of a haplotype-specific region located on chromosome 16 where both parents are presumably heterozygous for the region leading to three different groups. (TIF) [file pgen.1008213.s004.tif]

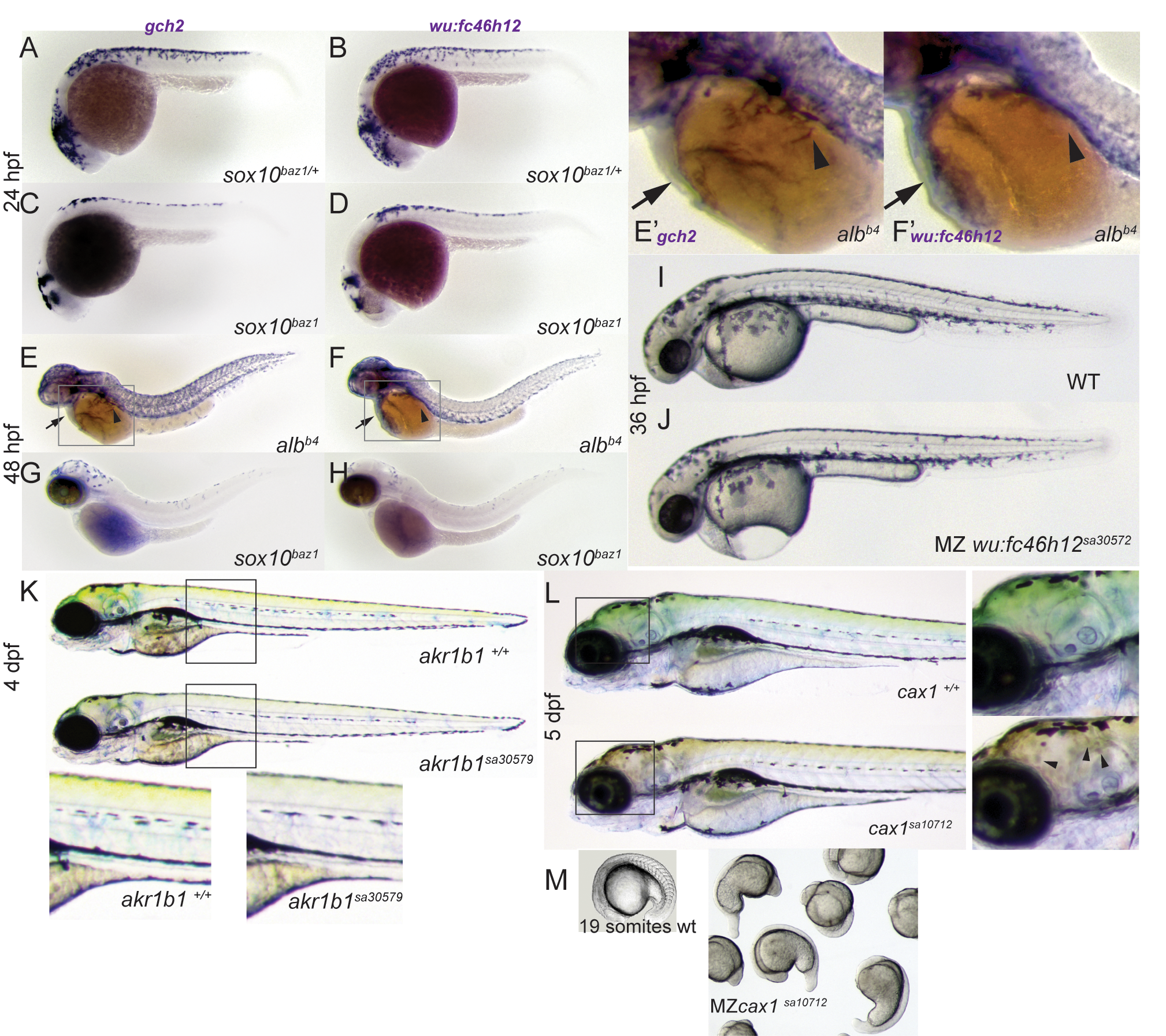

Supplement: S5 Fig — (A-H) Whole mount in situ analysis of wu:fc46h12 and gch2 as a pigment cell comparison. sox10baz/+ heterozygotes embryos as sibling controls (A-B) and mutant sox10baz1 embryos at 24 hpf (C-D). At 48 hpf in situs were carried out on albino embryos to serve as wild-type controls (E-F) with arrows indicating the heart and arrow heads the dorsal aorta. A blow up of this region can be found in E’-F’. (G-H) Expression of gch2 and wu:fc46h12 at 48 hpf in sox10baz1 mutants. I-J Wild-type and MZwu:fc46h12sa30587 embryos at 36 hpf with oedema around the forming heart (J). (K) Wild-type sibling and mutant akr1b1sa30579 at 4 dpf with mutant larvae presenting a reduction of yellow colour produced by xanthophores. Magnifications indicated with a black box. (L) Wild-type sibling and mutant cax1sa10712 larvae at 5 dpf. Close ups indicated by black boxes around the head show dull yellow colour and abnormal cell morphology in mutants (arrow head). (M) MZcax1sa10712 phenotype at 19 somite stage. (TIF) [file pgen.1008213.s005.tif]

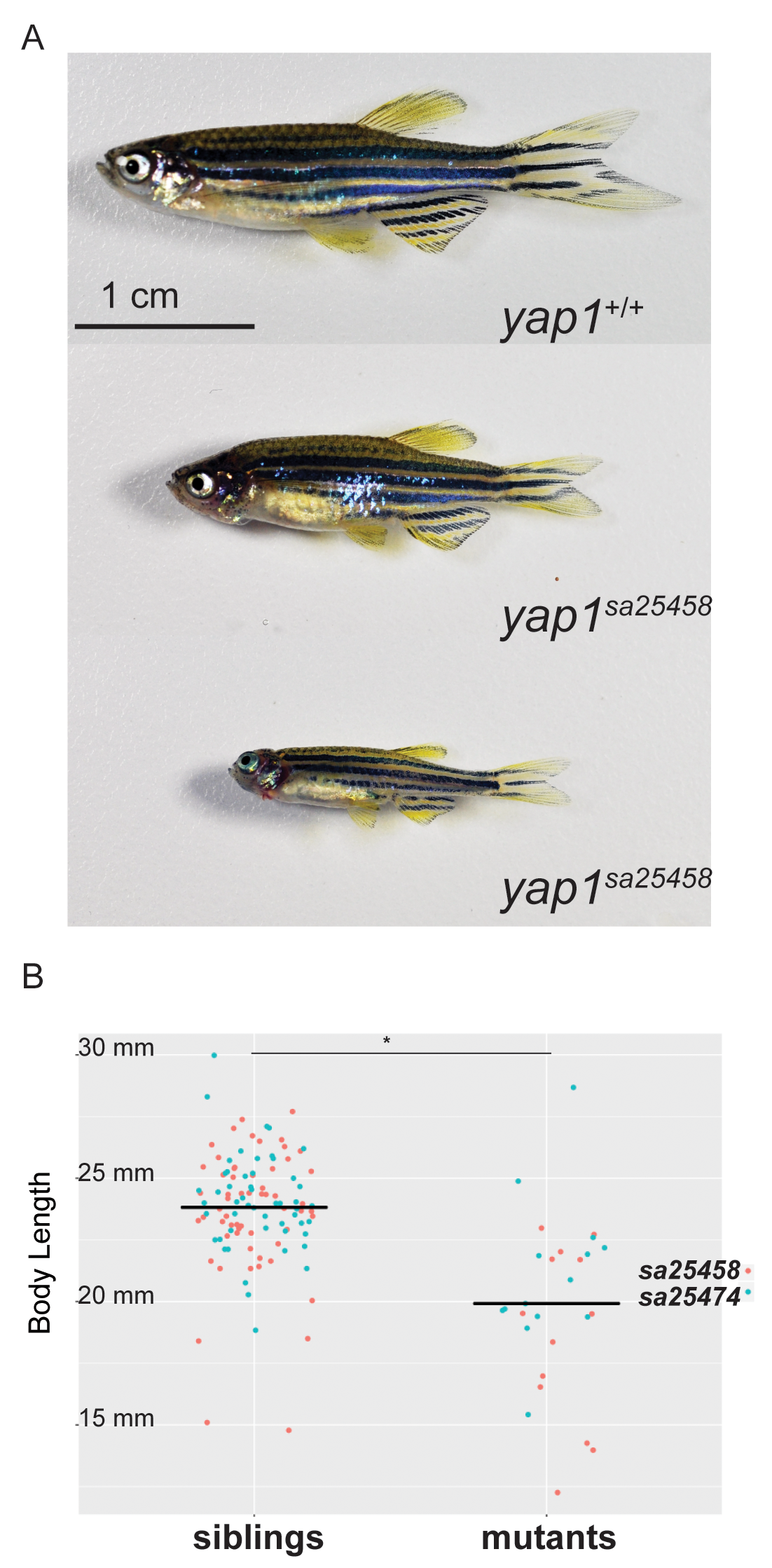

Supplement: S6 Fig — (A) Homozygous yap1 mutants are viable but present with a variation in size. (B) Quantification of size at two months of age with the corresponding genotypes for both yap1 alleles. A statistically significant difference with p-val <0.05 is indicated by “*”. (TIF) [file pgen.1008213.s006.tif]

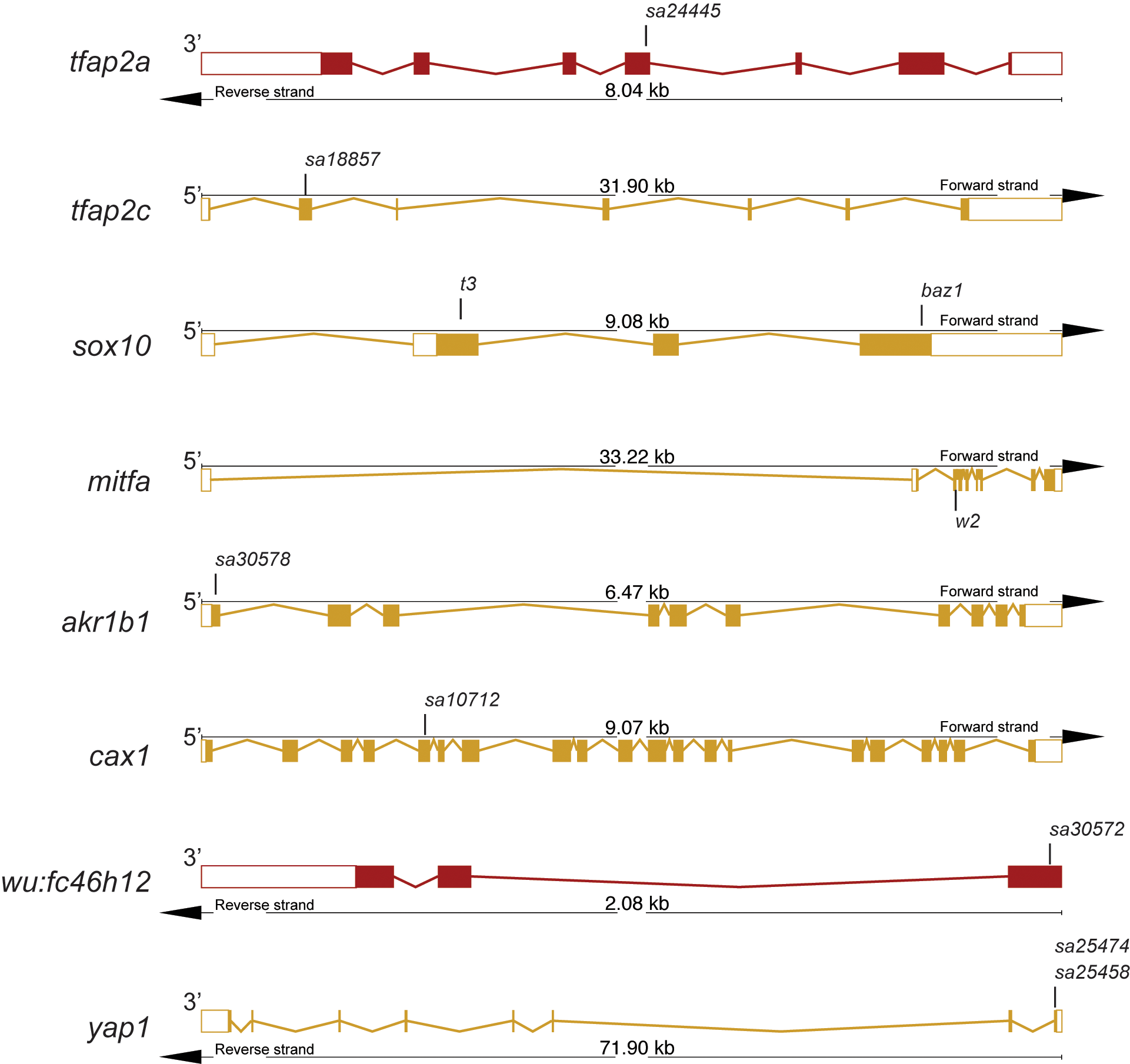

Supplement: S7 Fig — Filled boxes represent coding sequence, unfilled ones denote untranslated regions. (TIF) [file pgen.1008213.s007.tif]
